# Key characteristics of effective yoga interventions for managing osteoarthritis: a systematic review and meta-analysis

**DOI:** 10.1007/s00296-024-05652-y

**Published:** 2024-06-27

**Authors:** Isha Biswas, Gamze Nalbant, Sarah Lewis, Kaushik Chattopadhyay

**Affiliations:** 1 Lifespan and Population Health, School of Medicine, University of Nottingham, Nottingham, NG5 1PB UK; 2 The Nottingham Centre for Evidence-Based Healthcare: A JBI Centre of Excellence, Nottingham, NG5 1PB UK

**Keywords:** Meta-analysis, Osteoarthritis, Systematic review, Yoga

## Abstract

This systematic review aimed to synthesise the content, structure, and delivery characteristics of effective yoga interventions for managing osteoarthritis symptoms, including joint pain and joint function. JBI guidelines were followed. 17 databases were searched for randomised controlled trials (RCTs) assessing yoga’s effectiveness on osteoarthritis symptoms. Meta-analyses and a narrative synthesis were conducted to address the objective. The systematic review and meta-analysis included 18 and 16 articles (representing 16 and 14 RCTs), respectively. Overall, the included studies had low methodological quality scores. 10 of 14 yoga interventions effectively reduced pain (standardised mean difference (SMD) − 0.70; 95% confidence interval (CI) − 1.08, − 0.32) and/or improved function (− 0.40; − 0.75, − 0.04). Notably, 8 effective interventions had centre-based (supervised, group) sessions, and 6 included additional home-based (unsupervised, individual) sessions. Effective interventions included 34 yogic poses (12 sitting, 10 standing, 8 supine, 4 prone), 8 breathing practices, and 3 meditation and relaxation practices. 8 interventions included yogic poses, and 7 also incorporated breathing practices and/or meditation and relaxation practices. 4 interventions included yogic pose modifications for osteoarthritis. The median duration of centre-based sessions was 8 weeks and each session was around 53 min, mostly delivered once a week. The median duration of home-based sessions was 10 weeks and each session was 30 min, usually instructed to practice 4 times a week. Given previous studies’ limitations, a high-quality long-term RCT should be conducted using synthesised findings of previous effective yoga interventions.

**Supplementary Information:**

The online version contains supplementary material available at 10.1007/s00296-024-05652-y.

## Introduction

Osteoarthritis is the most common type of arthritis among adults, affecting more than 500 million people, globally [1, 2]. It is a long-term degenerative condition of the joints, characterised by main symptoms including pain, stiffness, and difficulty in movement [1]. It commonly affects the joints of the knees, hips, hands, and spine [1]. Osteoarthritis poses tremendous health (physical and psychological), social, and economic implications for the affected individual and society, severely affecting the individual’s self-efficacy and quality of life [3–6]. There is no cure for osteoarthritis, however, it can be managed [7]. The main aim of osteoarthritis management is to minimise joint pain and loss of function [7]*.* The traditional approaches to managing the symptoms rely mainly on pharmacological options (e.g., Non-Steroidal Anti-Inflammatory Drugs (NSAIDs)) along with non-pharmacological approaches (e.g., moderate-to-high intensity exercises) [8]. However, side effects of long-term usage of pharmacological options (e.g., gastrointestinal toxicity), limited adherence to non-pharmacological approaches (due to exercise-related injuries), and costs associated with both approaches (e.g., treatment-related costs, equipment costs for exercise) are some of the reasons which potentially limit their use among individuals with osteoarthritis [1, 9].

Yoga, a non-pharmacological treatment approach, has been recommended for osteoarthritis by an international osteoarthritis clinical guideline in 2019 [10] and some studies have also proposed yoga as a beneficial practice for people with arthritis [11, 12]. The ancient practice of yoga originated in the Indian subcontinent and imparts a sense of well-being of the body and mind [13]. Yoga philosophy and practice were first described by Patanjali in the classic text *Yoga Sutras* [14]*.* The multi-factorial approach of yoga includes components such as yogic poses (asana), breathing practices (pranayama), and meditation (dhyana) and relaxation practices, along with a moderated lifestyle [14]. Yoga practice generally begins with slow movement sequences to increase blood flow and warm up muscles, followed by holding certain yogic poses (e.g., extension, rotation) that engage the muscles in contraction [15, 16]. Movement of joints increases flexibility whereas standing poses improve balance and coordination by strengthening major muscle groups (e.g., hamstring muscles and quads), potentially reducing pain and improving function [17–19]. The worldwide popularity of yoga is rising, with nearly 300 million people across the world involved in its practice [20]. Generally, yoga is easy to learn with low risk involved, demands a low-to-moderate level of supervision, is inexpensive to maintain because of the minimal equipment requirement, and can be practised indoors and outdoors [21–23].

Existing systematic reviews and meta-analyses have reported the beneficial effects of yoga interventions on osteoarthritis symptoms, such as reduced pain and improved function [24–30]. These reviews have included randomised controlled trials (RCTs) [24–30], and in one review, also other study designs [29]. A systematic review and meta-analysis of 20 RCTs and 2 case series on knee and hip osteoarthritis showed that yoga significantly reduced pain scores (mean difference (MD) − 1.82, 95% confidence interval (CI) − 2.96 to − 0.67) and improved physical function scores (− 6.07, − 9.75 to − 2.39) compared to no intervention or usual care [29]. No adverse events related to yoga were reported [24–30]. However, all the above-mentioned systematic reviews have only described or reported but not synthesised the content, structure, and delivery characteristics of yoga interventions to manage osteoarthritis [24–30]. Therefore, this systematic review and meta-analysis aimed to fill this gap in the existing literature by narratively synthesising the content, structure, and delivery characteristics of effective yoga interventions for managing osteoarthritis symptoms. The intention was to facilitate the identification of the key features of effective yoga interventions for osteoarthritis, which could be combined for use in subsequent trials to test the effects of this yoga intervention on osteoarthritis patients.

## Methods

This systematic review followed the JBI methodology for systematic reviews of effectiveness and the Preferred Reporting Items for Systematic Reviews and Meta-analyses (PRISMA) guidelines [31, 32]. This review was conducted according to a priori published protocol [33] and registered with PROSPERO (CRD42022298155).

### Inclusion criteria

#### Population

We included studies conducted among adults (aged ≥ 18 years) diagnosed with osteoarthritis of one or more joints. No restrictions were applied regarding the diagnostic criteria of osteoarthritis; diagnoses based on physical examination, radiographic and MRI findings, and/or arthroscopy were included*.*

#### Intervention

Studies reporting at least one of the major components of yoga, namely, asana (yogic poses), pranayama (breathing practices), and dhyana (meditation) and relaxation practices were included. There were no restrictions on the type, frequency, duration, and delivery mode of the yoga intervention. Studies were excluded if they did not explicitly label the intervention as yoga.

#### Comparator

Studies comparing yoga interventions with no intervention, sham intervention, non-pharmaceutical intervention (e.g., diet, physical activity, and educational intervention), or pharmaceutical intervention (e.g., NSAIDs) were included*.* Studies with only a head-to-head comparison of two or more yoga interventions (i.e., different in terms of content, structure, or delivery characteristics) were excluded.

#### Outcome

We included studies that assessed the core outcomes of osteoarthritis, i.e., pain and/or function, as recommended in several guidelines [34–38]. Pain assessed using any scale (e.g., Visual Analogue Scale (VAS) and Numeric Rating Scale (NRS)) and function assessed using any scale (e.g., Arthritis Impact Measurement Scale (AIMS), including any joint-specific scale such as Foot and Ankle Ability Measure (FAAM), Knee Injury and Osteoarthritis Outcome Score (KOOS), and Hip Disability and Osteoarthritis Outcomes Survey (HOOS)) was eligible [34, 35].

### Study design

Only RCTs were included in the review, taking into account the feasibility and practical aspects of the research as well as the hierarchy of study designs.

### Data sources and search strategies

The following 13 databases were searched to find published studies from their inception dates to 22 September 2023: (1) MEDLINE (Ovid), (2) EMBASE (Ovid), (3) PsycInfo (Ovid), (4) CINAHL (EBSCOHost), (5) Cochrane Central Register of Controlled Trials (CENTRAL), (6) Allied and Complementary Medicine (AMED) (Ovid), (7) SPORTDiscus (EBSCOhost), (8) Web of Science (Clarivate Analytics), (9) Turning Research Into Practice (TRIP), (10) AYUSH Research Portal (http://ayushportal.nic.in/, accessed 22 September 2023), (11) A Bibliography of Indian Medicine (ABIM) (http://indianmedicine.eldoc.ub.rug.nl/, accessed 22 September 2023), (12) CAM-QUEST (https://www.cam-quest.org/en, accessed 22 September 2023), and (13) Physiotherapy Evidence Database (PeDro). Unpublished studies were searched using (1) OpenGrey (from 1997), (2) EthOS (from 1925), (3) ProQuest Dissertations and Theses (from 1980), and (4) DART-Europe-e-theses portal (from 1999). No language restrictions were applied. The search strategies were developed based on the following and in consultation with a Research Librarian at the University of Nottingham (UK): (1) the yoga component was based on a previous relevant systematic review [39], (2) the osteoarthritis component was based on the search strategies reported in the UK’s National Institute for Health and Care Excellence (NICE) guidelines for osteoarthritis management [40] and existing Cochrane systematic reviews on osteoarthritis [41, 42], and (3) the pre-designed search filters for RCTs were used [43–45]. All the search strategies are detailed in the supplementary file (Appendix 1). The reference list of all the included studies and relevant previous systematic reviews was screened for additional studies.

### Study screening and selection

Following the searches, all the identified citations were collated and uploaded to Endnote X9 (Clarivate Analytics, PA, USA) [46], and duplicate citations were removed. The remaining records were uploaded into Rayyan (Qatar Computing Research Institute [Data Analytics], Doha, Qatar) [47] and titles and abstracts were screened for eligibility by two independent reviewers (IB and GN). Studies identified as potentially eligible or those without an abstract had their full texts retrieved. The full texts of the studies were assessed for eligibility by two independent reviewers. Full-text studies that did not meet the inclusion criteria were excluded and the reasons for exclusion were reported (Appendix 2). Any disagreements that arose between the two reviewers were resolved through discussion. If a consensus was not reached, a third reviewer was consulted (SL/KC).

### Assessment of methodological quality

The included studies were critically assessed using the standardised critical appraisal tool developed by JBI for RCTs, by two independent reviewers (IB and GN), assigning a score as met (Y), not met (N), unclear (U) or not applicable (n/a) [31]. The two reviewers independently assessed each criterion and commented on it. Any disagreements that arose between the two reviewers were resolved through discussion. If a consensus was not reached, then a third reviewer was involved (SL/KC). Regardless of methodological quality, all studies underwent data extraction and synthesis.

### Data extraction

Data were extracted from the included studies using a pre-developed and pre-tested data extraction form, by two independent reviewers (IB and GN). If consensus was not reached through discussion, a third reviewer (SL/KC) was consulted. For both the symptoms (pain and function), the authors extracted the end-of-intervention data [34, 48]. Where this time point was not reported, data from the time point closest to the end of the intervention were extracted. Intention-to-treat (ITT) data were preferred compared to per-protocol data [49, 50]. Post-intervention data were extracted in preference to change from baseline data (i.e., post-intervention score − baseline score). Percentage change from baseline was not extracted, as it is highly sensitive to change in variance, and it also fails to protect from baseline imbalances, leading to non-normally distributed outcome data [51].

In the included studies, pain and function were reported as continuous data and so mean and standard deviation (SD) were extracted. Where no SDs were available, they were calculated from standard error (SE) or 95% CI using the formula from the Cochrane Handbook [52]. Where mean and SD for more than one intervention or control group were reported, the combined sample size, mean, and SD were calculated using the formulae in the Cochrane Handbook [52]. The corresponding authors of studies were contacted by e-mail (two times per author) to obtain missing or unclear data.

### Data synthesis

Considering the errors in how authors analyse and report yoga interventions to be effective in studies (e.g., conducting pre-post analysis of outcomes within study arms but no comparative analysis between study arms), meta-analyses were conducted for yoga vs. any comparator to determine the true effectiveness of each included yoga intervention for both the outcomes—pain and function. The meta-analyses were conducted using Review Manager 5.4.1 (Copenhagen, The Nordic Cochrane Centre, The Cochrane Collaboration) [53]. Random-effects meta-analyses were conducted due to the heterogeneous nature of the yoga interventions. Since the included studies used difference scales to report pain and function measures, standardised mean differences (SMDs) with 95% CIs were calculated using forest plots.

A narrative synthesis of the identified effective yoga interventions from the meta-analyses for pain and function was conducted with the aid of tables and text, focusing on the content, structure, and delivery characteristics of the yoga interventions. Commonalities and differences of the yoga interventions effective for either or both the outcomes were synthesised. The Sanskrit and English names of all the yogic components used in the effective interventions and the number of RCTs using these practices were tabulated.

## Results

### Study selection

**Fig. 1:**
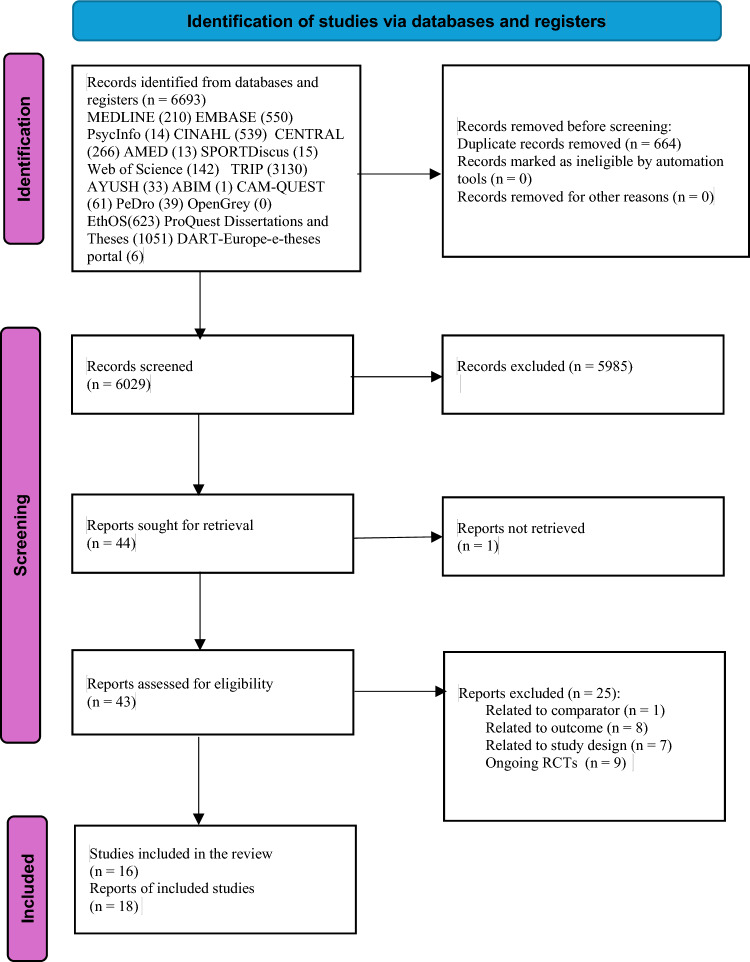
PRISMA flow diagram for included studies from searches of databases and registers only

6693 records were identified through the literature search. After removing duplicate records and title and abstract screening, 44 articles were retrieved for full-text screening. 18 articles were included in this systematic review representing 16 studies (RCTs) and 1402 participants [54–71]. 3 articles described the same RCT, providing data on different outcomes and therefore, were included as a single study in this review [55–57]. The study selection process is detailed in the PRISMA flowchart as shown in Fig. 1. No additional articles were identified from citation searching. The list of articles ineligible following the full-text review and ongoing RCTs identified from trial registries are presented in the supplementary file (Appendix 2).

### Description of the included studies

9 studies were conducted in the USA [54, 58–62, 66, 67, 70], 4 studies in India [55–57, 63, 64, 68], 1 study in Canada [65], 1 study in Iran [69], and 1 study in Australia [71]. The sample size of the studies ranged from 18 to 250. Studies recruited participants aged between 18 and 90 years and where reported, the mean age varied from 57 to 79 years. 13 studies included male and female participants and the remaining 3 recruited only female participants [58, 64, 65]. Where exact numbers were reported, female and male participants were 1049 and 333, respectively. 5 studies included participants with osteoarthritis of one or more lower extremity joints, including knee, hip, ankle or feet [59, 61, 62, 66, 67] and 9 studies included specifically knee osteoarthritis [55–58, 60, 63–65, 68, 69, 71], 1 on hand osteoarthritis [54], and the affected joint was unclear in another study [70]. Where reported, the duration of osteoarthritis amongst participants ranged from more than a month [69] to more than 2 years [55–57]. Of 16 studies, 7 reported the use of pain medications among the participants [58, 61, 65–67, 70, 71]. In 4 of 7 studies, it was unclear if the participants used pain medications before and/or during the trial [58, 61, 66, 67]. Where reported, 25–63% of participants used pain medications [61, 66]. 3 of 7 studies explicitly reported the use of pain medications among participants before the trial [65, 70, 71], and where specified, this ranged from 61 to 73% [65, 71]. In addition, 3 of 16 studies reported the use of non-pharmacological pain management (using physical therapy) before the trial, amongst 40%, 61%, and 78% of the participants, respectively [61, 70, 71]. Tables 1 and 2 report the characteristics of the studies and the details of the yoga interventions, respectively.

**Table 1:** Characteristics of included studies

Author and year	Country	Major inclusion criteria, including diagnosis	Population characteristics (age in years (M ± SD), sex(F/M), joint affected, duration of OA, any other therapy/medication for OA)					Sample size (n)	Intervention (I)	Comparator (C)
			Age in years (M ± SD)/M (95% CI)	Sex (F/M)	Joint affected	Duration of OA	Any other therapy/medication for OA			
Garfinkel, 1994 [54]	USA	Pain and/or stiffness in the hands Diagnosis: ACR guidelines	I = NR C = NR	Both sexes, (14/11)	Hands	NR	NR	25 (I = 14, C = 11)	I = Yoga program (yoga and relaxation techniques) and health education on OA	C = ’Largely’ drug-based treatment program
Ebnezar, 2011, 2012a, 2012b [55–57]	India	Persistent, moderate to severe walking pain for 3 months before recruitment Kellegren and Lawrence radiologic grading of II–IV in X-rays taken within last 6 months Diagnosis: ACR guidelines	I = 59.56 ± 8.18, 59.56 ± 9.54 (Ebnezar 2012b) C = 59.42 ± 10.66	Both sexes, I:88/37 C:86/39	Knee	< 1 year: I = 62, C = 59 1–2 years: I = 39, C = 40 > 2 years I = 24, C = 26	NR	250 (I = 125, C = 125)	I = IYT practice [including lectures and counselling on yoga and health] + (physiotherapy + TENS + ultrasound)	C = 40 min of non-yogic physiotherapy exercises^a^ supervised by certified therapists + (physiotherapy + TENS + ultrasound)
Cheung, 2014 [58]	USA	Community-dwelling adults aged 65–90 years Symptomatic OA diagnosis for 6 or more months No previous yoga training Not currently participating in a supervised exercise programme Diagnosis: ACR guidelines	I = 71.90 (69.30,74.60) C = 71.90 (69.00, 75.00)	Females, 36	Knee	≥ 6 months	Medication for arthritis (NS)	36 (I = 18, C = 18)	I = Hatha yoga	C = Wait-list control (60 min session/week and 30 min/day four times a week at home)
Park, 2016 [59]	USA	Community-dwelling adults aged ≥ 65 years Self-reported joint pain caused by OA Pain for 3 or more months Diagnosis: by a board-certified nurse practitioner using the criteria-based on OA symptoms	All = 75.30 ± 7.50	Both sexes, (75/25)	One or more lower extremity joints	NR	NR	100 (I1 = 32, I2 = 20, C1 = 28, C2 = 20)	I1 = Sit ‘N’ Fit chair yoga (English) I2 = Sit ‘N’ Fit chair yoga (Spanish)	C1 = HEP (English) C2 = HEP (Spanish)
Cheung, 2017 [60]	USA	Community-dwelling adults aged ≥ 60 years Self-reported medical diagnosis of symptomatic OA for 6 or more months No previous training in any form of yoga Not currently participating in a supervised exercise programme Diagnosis: NR	I = 68.90 ± 7.70 C1 = 74.40 ± 7.50 C2 = 71.80 ± 8.00	Both sexes, (70/13)	Knee	≥ 6 months	NR	83 (I = 32, C1 = 28, C2 = 23)	I = Hatha yoga	C1 = ASE^b^ (centre-based supervised group sessions and home-based unsupervised individual sessions, for 8 weeks) C2 = Education attention for 8 weeks (education brochures from the Arthritis Foundation on how to manage OA pain, and physical activity and exercise for OA)
McCaffrey, 2017 [61]	USA	Community-dwelling adults aged ≥ 65 years Self-reported joint pain caused by OA Moderate chronic pain for 15 or more days per month for 3 or more months Diagnosis: by a nurse practitioner based on OA symptoms	I = 75.90 ± 8.20 C = 74.50 ± 6.50	Both sexes, (85/27) I = 66/19, C = 19/8	One or more lower extremity joints	NR	Pain medication n = 70 (unclear if before or during the trial) History of non-pharmacological pain management n = 45	112 (I = 85, C = 27)	I = Sit ‘N’ Fit Yoga program (chair yoga)	C = HEP (health education sessions, including social interaction through games, lecture and discussion, by a health educator regarding OA)
Park, 2017[62]	USA	Ages ≥ 65 years Self-reported joint pain Moderate chronic pain (≥ 4 on a pain bother scale at least 15 days per month for ≥ 3 months Diagnosis: by a geriatric nurse practitioner	I** = **75.90 ± 8.20 C = 74.50 ± 6.50	Both sexes, (I:44/19, C:41/8)	One or more lower extremity joints	NR	NR	112 (I = 63, C = 49)	I = Sit ‘N’ Fit chair yoga	C = HEP (45-min health education sessions by a healthcare provider on OA)
Deepeshwar, 2018 [63]	India	OA > 3 months Diagnosis: by a physician	I = 59.80 ± 10.21 C = 61.07 ± 9.17	Both sexes, (I: 25/6, C: 25/10)	Knee	> 3 months	NR	66 (I = 31, C = 35)	I = IAYT	C = Conventional treatment without any form of yoga intervention
Kaur, 2018 [64]	India	Diagnosed with mild or moderate OA Not advised for surgery (viz. early OA) Diagnosis: ACR criteria for classification of idiopathic knee OA	I = 52.42 ± 4.60 C = 54.23 ± 4.80	Females, 83	Knee	NR	NR	83 (I = 43, C = 40)	I = Distribution of SIM + intervention package (group training through lectures and demonstrations (yoga asana & guided meditation)) one-to-one training through demonstration	C = Distribution of SIM
Kuntz, 2018 [65]	Canada	Ages ≥ 50 years Diagnosis: ACR guidelines	I = 65.50 ± 5.60 C1 = 63.70 ± 8.90 C2 = 71.10 ± 9.30	Females, 31	Knee	NR	Pain medications n = 19 (during the trial)	31 (I = 10, C1 = 11, C2 = 10)	I = Yoga exercise	C1 = Traditional exercise (knee strengthening and aerobic warm-up, balance exercises, and stretching C2 = No exercise (group-based, guided meditative relaxation classes led by a certified yoga-instructor)
Zacharia, 2018 [66]	USA	Community-dwelling adults aged 40–64 years Insufficiently active (< 30 min a day of moderate activity, five days a week, or < 150 min of moderate activity a week) or sedentary Diagnosis: NR	All = 57 ± 4.10 not reported separately for I/C	Both sexes, not reported separately for I/C	Lower limb joints (hip, knee, ankle, or feet)	> 6 months	Pain medications n = 5 (unclear if before or during the trial)	20 (I = 10, C = 10)	I = Hatha Yoga Program (Phase-1) + relapse Prevention programme (Phase-2)	C = No intervention
McCaffrey, 2019 [67]	USA	Ages ≥ 62 years Reported OA- associated pain Chronic pain at least 15 days of the month for ≥ 3 months Diagnosis: by a nurse practitioner	All = 78.80 ± 8.90, I** = **79.00 ± 2.50 C = 78.00 ± 2.10	Both sexes, (I: 5/4, C: 5/4)	Lower extremity joints (hip, knee, other LEs)	NR	I = Tylenol (acetaminophen), tramadol, ibuprofen n = 3 C = Tylenol (acetaminophen), ibuprofen n = 5 (unclear if before or during the trial)	18 (I = 9, C = 9)	I = Chair yoga (based on traditional Hatha yoga postures)	C = Chair exercise^c^
Vaghela, 2020 [68]	India	-Ages 40–80 years -Diagnosis: clinical ACR criteria	I** = **56.58 ± 10.12 C = 54.27 ± 8.44	Both sexes, (I:28/15, C:30/10)	Knee	NR	NR	83 (I = 43, C = 40)	I = Conventional physiotherapy + yoga therapy	C = Conventional physiotherapy: 1. Transelectrical nerve Stimulation (10 min) 2. Isometrics quadriceps exercise 3. Straight leg‑raising exercise in supine 4. Terminal knee extension or vastus medialis oblique strengthening exercise in supine and high sitting 5. Straight leg abduction exercise in side lying
Bokaeian, 2021 [69]	Iran	Ages 45–76 years Knee pain of 30 or greater on the 100-mm VAS Diagnosis: Unilateral or bilateral tibiofemoral joint OA of grades 2–3 based on the Kellgren–Lawrence grading system	I = 54.90 ± 5.00 C1 = 57.00 ± 4.90 C2 = 56.70 ± 4.70	Both sexes, (45/14) (I: 16/6, C1: 15/4, C2: 14/4)	Knee	Knee pain > 1 month	NR	59 (I = 22, C1 = 19, C2 = 18)	I = Yoga exercises and medial-thrust gait (YogaMT) training	C1 = Knee muscle strengthening C2 = Treadmill walking
Park, 2021 [70]	USA	Ages ≥ 60 years Moderate chronic OA pain of any joint for ≥ 15 days per month for ≥ 3 months Diagnosis: NR	All = 75.30 ± 7.50, not reported separately for I/C	Both sexes, (85/27)	NR	NR	Current pain medication n = 71 History of non-pharmacological pain management n = 68	112 (I = 47, C = 65)	I = Chair yoga	C = Health education programme consisting of information on OA by a healthcare provider
Bennell, 2022 [71]	Australia	Ages ≥ 45 years Activity-related knee pain for at least 3 months Average walking pain score of ≥ 4 on an 11-point numerical rating scale over the previous week No knee morning stiffness lasting ≥ 30 min) Diagnosis: NICE clinical criteria*	I = 62.80 ± 8.20 C = 61.80 ± 7.20	Both sexes, (I: 70/37, C: 78/27)	Knee	Knee pain > 3 months	Current pain medications: I&C = NSAIDs, acetaminophen, topical NSAIDs, oral corticosteroids and oral opioids History of treatment in last 3 months: Massage/manual therapy Gait aid Thermal therapy/electrotherapy Orthotics, arch supports, or wedging in shoes Knee braces Land-based and water exercises Joint injections Acupuncture Knee surgery	212 (I = 107, C = 105)	I = Online yoga programme plus online education	C = 24-weeks unlimited access to a customized trial website containing downloadable educational covering understanding OA, treatment options, exercise and physical activity, weight loss, understanding and managing pain, sleep, and patient stories

Only yoga-related interventions were mentioned under intervention, and no intervention or any other active interventions were mentioned under comparator. OA: osteoarthritis, ACR: American College of Rheumatology, NR: Not reported, ADL: Activities of daily living, IYT: Integrated Yoga Therapy, TENS: Transcutaneous Electrical Stimulation, HEP: Health Education Programme, IAYT: Integrative Approach for Yoga Therapy, SIM: Self-Instruction Manual, NSAIDs: Non-Steroidal Anti-Inflammatory Drug.

^a^Physiotherapy exercises included loosening and strengthening practices for upper and lower limb joints, rest, specific knee practices, and supine rest followed by light music.

^b^Weekly group sessions by an instructor for 8 weeks (15 min mild aerobic exercise and 30 min of strengthening exercises including both isometric (without moving the joints) and isotonic (moving the joints) exercises. Home practice: aerobic exercise for 15–30 min/day, four times/week, and the strengthening exercises for 30 min/day, two times/week on non-consecutive days.

^c^Chair exercise consisting of progressive resistive exercises incorporating body weight and/or external resistance using cuff weights, resistance bands, and balls, NICE clinical criteria: (age ≥ 45 years, activity-related knee pain, and no knee morning stiffness lasting ≥ 30 min)

**Table 2:** Intervention details (key features of content, structure and delivery characteristics) of the included RCTs

Author and year	Intervention development	Intervention duration	Yoga sessions: content			Yoga sessions: structure (duration and frequency)	Yoga sessions: delivery characteristics (*location, settings, instructor, monitoring and adherence)*	Extra features
			Asana	Pranayama	Dhyana and relaxation			
*Garfinkel, 1994*[54]	*Based on a literature review on OA, and the study and practice of hatha yoga consisting of supervised yoga and relaxation techniques and patient education*	*10 weeks*	*Parvatasana (mountain pose)*	*Attention to respiration*	*NR*	*I* = *60 min session/weekX10 weeks (1st and 10th session devoted to pre and post testing)*	*Centre-based group sessions delivered and supervised by a health educator and yoga teacher*	*Written instructions of educational materials provided weekly* *Group discussion, supportive encouragement and general QnA* *No information on encouragement for yoga practice after the intervention*
*Ebnezar, 2011,2012a, 2012b*[55–57]	*Developed from the traditional yoga scriptures (Patanjali yoga sutras, yoga vasishta, and Upanishads)*	*12 weeks*	*Yogasanas-10 min* *Standing asanas:* * 1. Tadasana (mountain pose)* * 2. Ardha Kati Chakrasana (lateral arc pose)* * 3. Ardha Chakrasana (half wheel pose)* * 4. Prasarita Padahastasana (wide-legged standing forward bend pose)* *Lying asanas:* * 1. Bhujangasana (Cobra pose)* * 2. Shalabasana (locust pose)* * 3. Viparita Karani (legs-up-the-wall pose)* * 4. Dhanurasana (bow pose) (reported only in Ebnezar 2012a)*	*Nadishuddhi Pranayama (alternate nostril breathing)-3 min*	*1. OM meditation-2 min* *2. Deep relaxation technique-5 min* *3. Quick relaxation technique–3 min* *4. Instant relaxation technique-2 min*	*I* = *40 min IYT (25 min of asana, pranayama and dhyana and relaxation practices)* + *20 min (physiotherapy with TENS and ultrasound)/6 days/week X 2 weeks* + *40 min IYT/day at home daily X 12 weeks*	*Centre-based group sessions at a hospital basement and home-based individual sessions* *Sessions delivered and supervised by certified therapists* *Home practice documented in diaries, telephone calls once in 3 days and a weekly review to monitor home practice*	*Other practices in the IYT session included loosening practices of foot and ankle, knee, hip and waist, upper limbs, and neck, strengthening exercises* *Lectures and counselling on yogic concepts of health and disease, yama, niyama, bhakti yoga, Jnana yoga and karma yoga were included in the yoga module* *Sessions for 40 min daily (6 days/week) after physiotherapy (20 min) for 2 weeks*
*Cheung, 2014*[58]	*Designed by a panel of five certified yoga teachers who had experiences teaching older adults* *Reviewed by 2 yoga researchers and a yoga master*	*8 weeks*	*Asanas in the seated, supine, and standing positions with the following key postures:* * 1. Tadasana (mountain pose)* * 2. Virabhadrasana I (warrior I pose) and Virabhadrasana II (warrior II pose)* * 3. Vrksasana (tree pose* * 4. Utkatasana (chair pose)* * 5. Sukhasana (easy seated pose)* * 6. Baddha Konasana (bound angle pose)* * 7. Upavistha Konasana (open angle pose)* * 8. Ardha Shalabhasana (half locust pose)* * 9. Setu Bandha Sarvangasana (bridge pose)* * 10. Uttanasana (standing forward fold reclining pose)* * 11. Hamstring stretches* * 12.Reclining twist*	*NR*	*Relaxation poses*	*I* = *60 min/weekX8 weeks* + *30 min/dayX4 times a week of yoga practice at home for 8 weeks*	*Centre-based group and home-based individual sessions at a yoga studio* *Sessions delivered and supervised by a registered yoga teacher* *Handouts of illustrated yoga poses were distributed for home practice* *Log sheet used for monitoring home practice*	*Poses with static stretching, balance, and strength exercises* *Mats, blocks, straps, blankets, and chair used during class, and poses were modified as per individual needs*
*Park, 2016*[59]	*Developed by a research team of health care providers with a yoga teacher who has taught yoga for more than 15 years, certified by the International Yoga Alliance*	*8 weeks*	*1. Body proper-20 min* *2. Virabhadrasana (warrior in the body pose)-5 min*	*Breath of life-10 min*	*Mind–body connection-10 min*	*I* = *45 minX2/weekX8 weeks*	*Centre-based group sessions delivered and supervised by a certified yoga instructor at community sites* *Participants were given a Sit ‘N’ Fit chair yoga manual with detailed instructions and pictures for home practice after 8 weeks*	*Chair used as a support for standing poses* *Each yoga cohort at each site had a maximum ratio of participants to instructor of 10:1* *CY manual translated to Spanish, for Spanish-speaking participants*
*Cheung, 2017*[60]	*Designed by a group of expert yoga teachers*	*8 weeks*	*Poses in seated, supine, prone and standing positions* * 1. Supta Baddha Konasana (reclining bound angle pose)* * 2. Ardha Salabhasana (half locust variation)* * 3. Janu Sirsanana (head to knee pose)* * 4. Uttanasana (standing forward fold pose)* * 5. Utkatasana (chair pose)* * 6. Tadasana (mountain pose)* * 7.Virabhadrasana I (warrior I pose)* * 8.Virabhadrasana II (warrior II pose)* * 9.Vrksasana (tree pose) variation* * 10. Reclining hamstrings stretch with hip opener with strap* * 11.Reclining twist*	*NR*	*Relaxation pose*	*I* = *45 min/weekX8 weeks* + *30 min/dayX4 times a week of yoga practice at home for 8 weeks*	*Centre-based group and home-based individual sessions* *Centre-based sessions delivered and supervised by certified instructor* *Handouts with pictures and written instructions were distributed for home practice* *Home practice monitored using video recordings, and self-recorded yoga/exercise log sheets*	*Use of props: yoga mats, blocks, straps, blankets, and chairs* *Poses were modified when needed*
McCaffrey, 2017[61]	Created by a master yoga instructor and used in 2 pilot studies to determine feasibility and participant satisfaction Sit “N” Fit is based on the foundation of Iyengar Hatha Yoga	8 weeks	Body Proper-20 min Warrior in the Body-5 min: 1. Saaras Pakshi Asana (stork pose) 2. Bhujangasana (cobra pose) 3. Vrksasana (tree pose) 4. Salabhasana (locust pose) 5. Ardha Chandrasana (half-moon pose) 6. Bharmanasana (table pose)	Breath of Life-10 min (1 breathing technique per class) 1. Diaphragmatic breathing 2. Nadi Shodhana Pranayama (alternate nostril breathing) 3. Ocean breath (Ujjayi breath)	Mind–Body Connection-10 min 1. Tense and relax 2. Total body guided relaxation 3. Guided visualisation	I = 45 minX2/week X8 weeks	Centre-based group sessions delivered and supervised by certified yoga instructors Manuals with instructions and pictures was provided for home practice Post intervention, participants were asked to continue to do the yoga program at home at least twice a week and to document home practice	Chair used as a support for standing poses
*Park, 2017*[62]	*Developed by a master yoga instructor with more than 20 years of experience with older adults. It was built on the Iyengar Hatha yoga technique, recommended by the NCCIH*	*8 weeks*	*NR*	*NR*	*NR*	*I* = *45 minX2/week X8 weeks*	*Centre-based group sessions at a senior housing facility and a senior day centre delivered and supervised by certified yoga instructors* *Post intervention, participants were given a manual with instructions and pictures for home practice* *Participants were asked to report frequency, duration, and components of home practice after 3 months*	*Chair was used* *Fidelity of yoga was ensured by the program developer*
*Deepeshwar, 2018*[63]	*Developed using a holistic approach to health management at physical, mental, emotional, and intellectual levels*	*1 week*	*Asanas (yoga postures) – 19 min* *Sitting – 6 min* * 1. Paschimottasana (seated forward bend pose)-3 rounds min)* * 2. Bhunamanasana (earth salutation pose)-3 rounds (3 min)* *Prone – 6 min* * 1. Bhujangasana (cobra pose)-3 rounds (3 min)* * 2. Salabhasana (locust pose) – 3 rounds (1 min)* * 3. Vipareetkarani (inverted pose)-2 min* *Supine – 7 min* * 1. Setubandhasana (bridge pose)-2 rounds (1 min)* * 2. Markatasana (lumbar stretch pose)-2 rounds (1 min)*	*Total—32 min* *Pranayama (yoga breathing)—12 min* * 1. Vibhagıya Pranayama (sectional breathing)-3 rounds (3 min)* * 2. Nadıshuddhı Pranayama (alternate breathing)-9 rounds (3 min)* * 3. Brahamarı Pranayama (humming bee breathing)-9 rounds (3 min)* *4. Bhastrika Pranayama (bellows breathing)-9 rounds (3 min)* *Cooling pranayama—9 mins* * 1. Sıtali (rolling tongue breathing)-9 rounds (3 min)* * 2. Sitkarı (folded tongue breathing)-9 rounds (3 min)* * 3. Sadanta (clenched teeth breathing)-9 rounds (3 min)* *Kapalabhati (frontal brain cleansing breath)-5 min* *Breathing practices—6 min* * 1. Hands in and out breathing-5 rounds (2 min)* * 2. Hands stretch breathing- 5 rounds (2 min)* * 3. Ankle stretch breathing-5 rounds (2 min)*	*Total – 82 min* *Savasana (Corpse pose)-5 min* *Relaxation Techniques – 17 mins* * 1. Instant relaxation technique-2 min* * 2. Quick relaxation technique- 5 min* * 3. Deep relaxation technique- 10 min* *Cyclic meditation-30 min* *Om meditation and devotional sessions (prayers)-10 min* *Mind sound resonance technique-10 min* *Nadanusandhana (A,U,M and A-U-M Kara chanting)-10 min*	*I* = *133 minX2 times a dayX1 week*	*Centre-based group sessions delivered (supervised? unsupervised?) at a yoga centre of S-VYASA in Bangalore, India*	*Lectures and individual yogic counselling* *Loosening practices in standing – 6 min* * 1. Twisting- 5 rounds (2 min)* * 2. Side bending- 5 rounds (2 min) on each side* *Loosening practices in Sitting – 12 min* * 1. Knee cap tightening-, 5 ounds (2 min each), both legs* * 2. Passive Patella Movement (Up and Down, In and Out, Rotation)- 10 rounds (4 min) both legs* * 3. Knee bending- 5 rounds (2 min) each, both legs* *Loosening practices in supine – 6 min* * 1. Folded Leg Lumber Stretch (Left, Right, Both)- 5 rounds (2 min)* * 2. Cycling- 5 rounds (2 min) both legs* * 3. Straight Leg Raising (Left, Right and Both)- 5 rounds (2 min)* *Kriyas (cleansing techniques) everyday twice (morning and evening)—55 min* * 1. Jalaneti (nasal cleansing with water)-30 min* * 2. Vamanadhouti (internal cleansing by water)-15 min* * 3. Trataka (candle light gazing)-10 min*
*Kaur, 2018*[64]	*Developed in Punjabi (vernacular) language and circulated among yoga specialists for consensus validity. It was modified as per the feedback received*	*6 months*	*NR*	*NR*	*NR*	*I* = *60 minsX2/monthX2 months* + *60 min/monthX4 months*	*Centre-based group sessions (supervised) through lectures and demonstrations and home-based individual practice using self-instruction manuals* *Participants were asked to continue home practice during the intervention period*	*Joint conditioning exercises* *Participants required to attend six of the eight intervention sessions to be included in the analysis*
Kuntz 2018[65]	NR	12 weeks	Selected weight-bearing, static poses which included squats and lunges with varying foot, trunk, and arm positioning	None	Supine body awareness exercise at the start of the session Supine deliberate relaxation exercise at the end of the session	I = 60 minX3/week X12weeks	*Centre-based group sessions delivered and supervised* by a certified, trained yoga instructor Session attendance and program adherence were monitored	Careful attention paid to the ideal alignment of the leg throughout the exercises Exercise difficulty was progressively increased
Zacharia, 2018[66]	NR	8 weeks	14 Hatha yoga poses	NR	Relaxation using restorative poses-10 min 1. Viparita Karani (leg-up-the-wall pose) 2. Savasana (corpse pose)	1st phase: I = 60 minX2/weekX8 weeks 2nd phase: I = 120 min/weekX4 weeks	*Centre-based group sessions through community classes, delivered and supervised by a certified yoga instructor* *Home-based individual practice using a yoga practice sheet with detailed instructions on an* information brochure Participants were encouraged to continue yoga practice at home through weekly emails and phone calls and text messages Yoga practice log was used to monitor home practice	For targeting social support, participants were encouraged to invite a family member or a friend to practice yoga with them The text messages included weekly tips on setting an effective yoga routine, and meet a weekly yoga target of 120 min/week The messages also included the names of yoga apps for smart phones, YouTube videos on practicing yoga at home, yoga classes currently conducted in the area, and long-term OA self-care tips
McCaffrey, 2019[67]	Developed based on traditional Hatha yoga postures	8 weeks	NR-25 min	NR-10 min	NR-10 min	I = 50 minX2/weekX8 weeks	Centre-based group sessions delivered and supervised by a certified yoga instructor certified by the National Yoga Alliance, conducted at a senior housing facility	Chair was used as a support
*Vaghela, 2020*[68]	*NR*	*4 weeks*	*Six asanas:* * 1. Tadasana (mountain pose)* * 2. Utthita Trikonasana (triangle pose)* * 3. Virabhadrasana (warrior pose)* * 4 .Dandasana (staff pose)* * 5. Supta Padangustasana (reclining hand-to-big-toe pose)* * 6. Baddha Konasana (bound angle pose)*	*None*	*None*	*I* = *30 minX3/weekX4 weeks*	*NR*	*Each asana consisted of ten repetitions with short intervals of rest*
Bokaeian, 2021[69]	NR	4 weeks	Goddess squat and warrior lunge exercises-20 min Goddess squat: 1. Level 1: hands on hips knees flex to 30° 2. Level 2: hands on hips knees flex to 60° 3. Level 3: shoulders flex to 90° elbows straight knees flex to 60° 4. Level 4: arms overhead knees flex to 80° Warrior lunge: 1. Level 1: hands on hips 2. Level 2: shoulders flex to 90° with elbows straight 3. Level 3: arms overhead 4. Level 4: arms overhead look up to the ceiling for an added challenge	None	None	I = 20 minsX3/weekX4 weeks	*Group sessions delivered and s*upervised by a physiotherapist	Level of difficulty of the goddess and warrior exercises was adjusted based on the Borg Perceived Exertion Scale Participants also received thermotherapy with a hot pack for 20 min
Park, 2021[70]	Developed by the research team that provided step-by-step instructions for continuing yoga at home	8 weeks	NR-25 min	NR-10 min	NR-10 min	I = 45 minX2/weekX8 weeks	Centre-based group sessions at a *senior housing facility or senior centre delivered and supervised by a yoga instructor and* home-based individual sessions provided using g*uided manuals*	Chair was used *Fidelity ensured by observing and assessing 20% of the sessions based on a standardised checklist developed by the research team*
*Bennell, 2022*[71]	*Designed by the researchers and a panel comprising 5 yoga therapists (registered with the International Association of Yoga Therapists or the Australasian Association of Yoga Therapists), 2 people with knee OA, and a physiotherapist with fitness leader qualifications and expertise in teaching yoga*	*12 weeks*	*Yoga postures to warm up the core and lower-extremity muscles-5 min* *Static and dynamic yoga postures intended to activate, strengthen, and stretch core and lower-extremity muscles-20 min* * 1. Knee to chest- Apanasana* * 2. Leg extensions supported squat- Utkata Konasana* * 3. Supported forward bend-Paschimottasana* * 4. Straddle seat-Upavistha Konasana* * 5. Straddle seat prep standing forward lunge* * 6. Leg lifts* * 7. Hip lifts* * 8. Marching one leg balance* * 9. Wide leg side bend* * 10. Standing side lunge-Skandasana* * 11. Knee hugs* * 12. Twisted pose* * 13. Wide leg forward bend (Prasarita Padottanasana)*	*NR*	*1. Cooldown of stretches* + *relaxation exercises-5 min* *2. Salamba Savasana (Supported rest)*	*I* = *30 minX3/weekX 12 weeks*	*Home-based individual unsupervised sessions delivered online* *Pre-recorded videos included a demonstration of yoga by a yoga instructor (a physiotherapist and yoga teacher)* *Weekly reminders and motivational emails sent to increase adherence*	*Video class instructor demonstrated various modifications and levels for each posture to ensure safety and feasibility* *12 different pre-recorded 30-min videos (1 video per week)* *Upon completing the program, yoga practice was recommended but optional*

NR: Not reported (Only the terms—Asana, Pranayama or Dhyana and Relaxation practices were mentioned but not no details provided) Text in *italics***:** Effective interventions for pain and/or function. None: No

Mention of any of the terms—asana, pranayama or dhyana and relaxation practices, OA:osteoarthritis, QnA: question and answer, IYT: Integrated Yoga Therapy, TENS: Transcutaneous Electrical Stimulation, NCCIH: National Centre for Complementary and Integrative Health

### Methodological quality of included studies

Table 3 reports the methodological quality of the included studies. Overall, the methodology was not adequately reported in the included studies, resulting in low methodological quality scores (total “yes” percentage ranging from 15 to 54%). In real practice, yoga providers delivering the yoga intervention cannot be blinded. So, the response to question 5 of the checklist was marked as N/A (not applicable) in our methodological assessment. Some of the major issues in these studies included: (1) inadequate reporting of the randomisation process used to assign participants to study arms and thus, it was unclear if true randomisation was used or not; (2) inadequate reporting of the allocation concealment process and thus, it was unclear if the allocation to study arms was concealed or not; (3) imbalance between the treatment groups at baseline; (4) inadequate reporting of blinding of participants and outcome assessors (could have been achieved through sham therapies); (5) inadequate reporting of whether the study arms were treated identically other than the intervention of interest; (6) insufficient analysis of the differences between study groups about loss to follow up and reasons for loss to follow up in case of incomplete follow up for the entire trial duration; (7) inadequate reporting of ITT analysis and its details (i.e., whether participants were analysed in the groups to which they were initially randomised); (8) inadequate reporting of the measurement process of outcomes (including adverse events) and thus, it was unclear if the outcomes were measured in the same way for study arms or not; (9) no or inadequate description of the number of raters who assessed outcomes or their training, hence making it unclear if outcomes were assessed in a reliable manner; and (10) issues in the statistical power analysis, unclear minimum clinically important difference for the sample size calculation, no information on assumptions of statistical tests used, and errors in statistical analysis and reporting (e.g., pre-post analysis and not between groups).

**Table 3:** Methodological quality assessment of the included studies

Study	Q1	Q2	Q3	Q4	Q5	Q6	Q7	Q8	Q9	Q10	Q11	Q12	Q13	Total % of “Y”
Garfinkel, 1994 [54]	U	U	U	U	N/A	U	Y	U	U	U	U	U	Y	15
Ebnezar, 2011 [55]	Y	U	Y	N	N/A	Y	Y	U	U	Y	U	U	Y	46
Ebnezar 2012a [56]	Y	U	Y	N	N/A	Y	Y	U	U	Y	U	U	Y	46
Ebnezar 2012b [57]	Y	U	Y	N	N/A	Y	Y	U	U	Y	U	U	Y	46
Cheung, 2014 [58]	Y	U	N	N	N/A	Y	U	Y	Y	U	U	N	Y	38
Park, 2016 [59]	Y	U	N	N	N/A	Y	U	U	N	Y	U	U	Y	31
Cheung, 2017 [60]	Y	U	Y	U	N/A	Y	U	U	Y	U	U	Y	Y	46
McCaffrey, 2017 [61]	Y	U	U	U	N/A	Y	U	Y	Y	Y	U	Y	Y	54
Park, 2017 [62]	Y	U	U	U	N/A	Y	U	Y	Y	Y	U	U	Y	46
Deepeshwar, 2018 [63]	U	U	Y	N	N/A	Y	Y	Y	U	Y	U	Y	Y	54
Kaur, 2018 [64]	U	U	Y	U	N/A	U	U	Y	N	U	U	U	Y	23
Kuntz, 2018 [65]	Y	U	N	U	N/A	Y	U	U	N	Y	U	U	Y	31
Zacharia, 2018 [66]	U	U	U	U	N/A	U	U	Y	U	U	U	Y	U	15
McCaffrey, 2019 [67]	Y	U	Y	U	N/A	Y	U	U	Y	U	U	N	Y	39
Vaghela, 2020 [68]	U	U	Y	U	N/A	U	U	N	N	Y	U	U	Y	23
Bokaeian, 2021 [69]	N	U	N	U	N/A	Y	Y	Y	Y	Y	U	U	Y	46
Park, 2021 [70]	Y	U	U	U	N/A	Y	U	Y	Y	Y	U	U	Y	46
Bennell, 2022 [71]	Y	Y	N	N	N/A	Y	U	Y	Y	U	U	Y	Y	54

In real practice, yoga providers delivering the yoga intervention cannot be blinded. So, answer to question 5 of the checklist was marked as N/A (not applicable). This tool uses a series of criteria that can be scored as being met (yes), not met (no), unclear or not applicable (n/a). Y = yes; N = no; U = unclear; NA = not applicable

JBI critical appraisal checklist for randomised controlled trials: Q1. Was true randomisation used for assignment of participants to treatment groups? Q2. Was allocation to treatment groups concealed? Q3. Were treatment groups similar at baseline? Q4. Were participants blind to treatment assignment? Q5. Were those delivering treatment blind to treatment assignment? Q6. Were outcomes assessors blind to treatment assignment? Q7. Were treatment groups treated identically other than the intervention of interest? Q8. Was follow-up complete and if not, were differences between groups in terms of their follow up adequately described and analysed? Q9. Were participants analysed in the groups to which they were randomised? Q10. Were outcomes measured in the same way for treatment groups? Q11. Were outcomes measured in a reliable way? Q12. Was appropriate statistical analysis used? Q13. Was the trial design appropriate, and any deviations from the standard RCT design (individual randomisation, parallel groups) accounted for in the conduct and analysis of the trial?

### Meta-analysis to determine effective studies

Of 16 studies included in this review, 2 could not be included in the meta-analysis because of insufficient data to calculate the mean and SDs for the yoga and control groups [61, 70]. Therefore, a total of 14 studies (16 articles) were included in the meta-analysis to identify the individual effective interventions for each symptom—pain (13 studies) and function (13 studies). These studies compared yoga interventions with pharmacological and non-pharmacological interventions [54–60, 62–69, 71].

#### Yoga vs comparator for pain

**Fig. 2:**
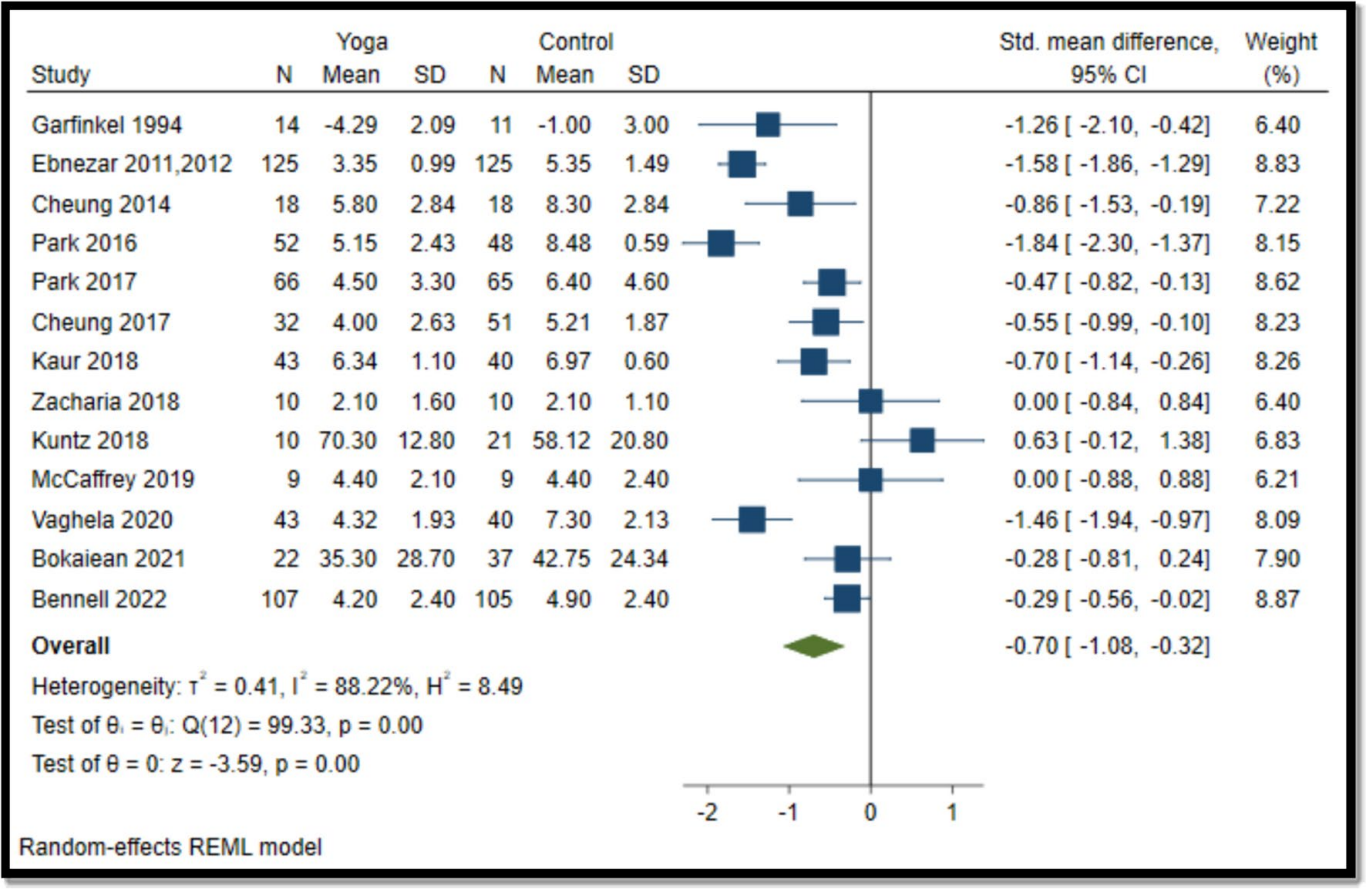
Forest plot for yoga vs comparator (pain). N, sample size; SD, standard deviation; CI, confidence interval

Overall, yoga interventions reduced pain compared to pharmacological and non-pharmacological interventions (SMD − 0.70; 95% CI − 1.08 to − 0.32) (Fig. 2).

#### Yoga vs comparator for function

**Fig. 3:**
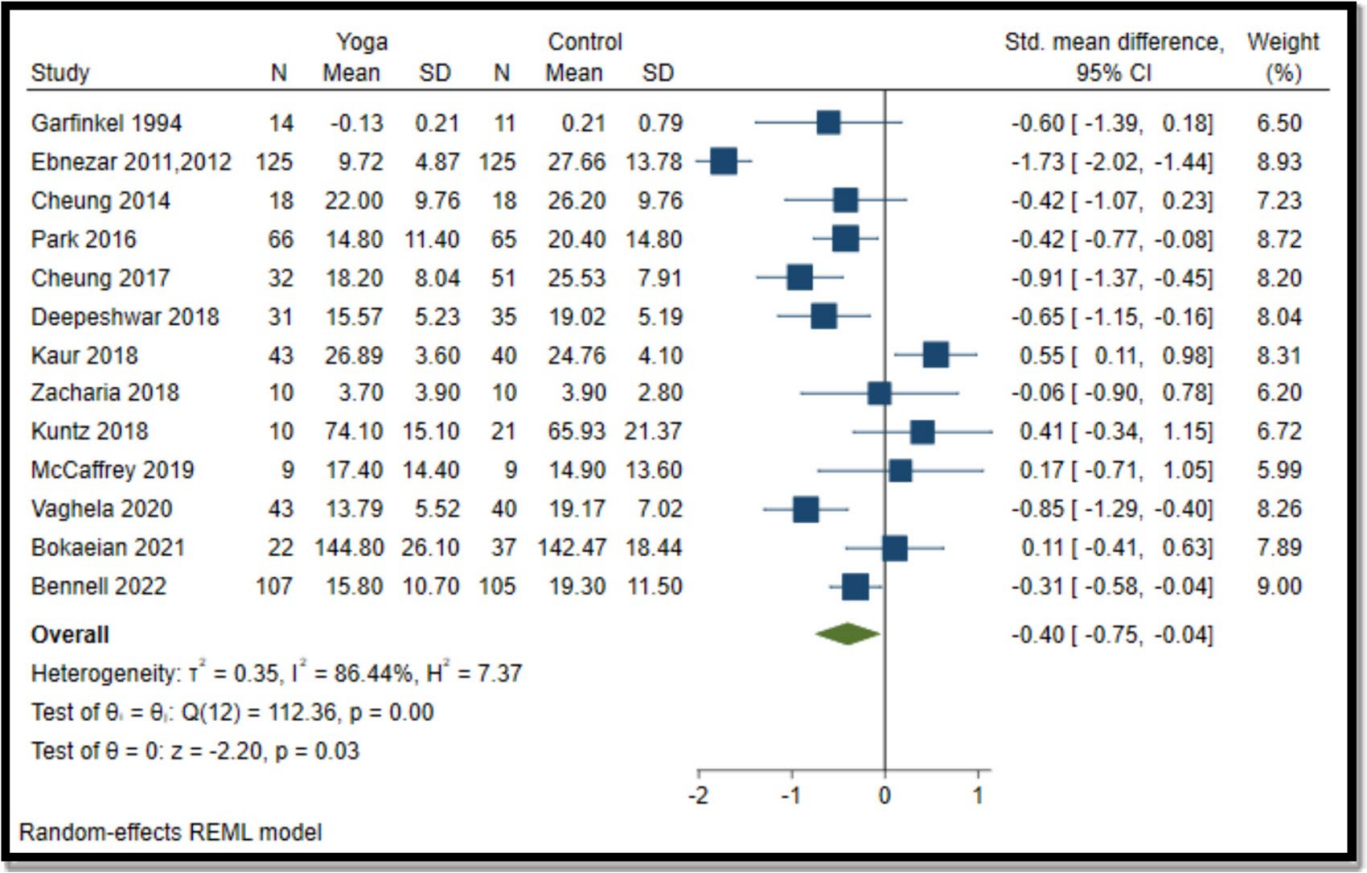
Forest plot for yoga vs comparator (function). N, sample size; SD, standard deviation; CI, confidence interval

Overall, yoga interventions were effective in improving function compared to pharmacological and non-pharmacological interventions (SMD − 0.40; − 0.75 to − 0.04) (Fig. 3).

Yoga interventions in 9 (out of 13) studies were effective in reducing pain (6 in knees [55–58, 60, 64, 68, 71], 2 in lower extremities [59, 62], and one in hand [54]. Yoga interventions in 6 (out of 13) studies were effective in improving function (5 in knees [55–57, 60, 63, 68, 71]) and one in the lower extremity [62]. Overall, 10 (out of 14) interventions were effective in reducing pain and/or improving function [54–58, 60, 62–64, 68, 71].

### Content, structure, and delivery characteristics of effective yoga interventions for pain and/or function

Notably, 6 of 10 effective interventions had centre-based (supervised, group) sessions with additional home-based (unsupervised, individual) sessions [55–60, 62, 64], 2 interventions were completely centre-based [54, 63] and one home-based [71]. Of the 2 completely centre-based interventions, one was supervised [54] and supervision detail was unclear in the other [63]. One study did not provide any details on intervention delivery [68]*.*

The content of effective yoga interventions was heterogeneous and included 34 different yogic poses (asana; 12 sitting, 10 standing, 8 supine, and 4 prone), 8 breathing practices (pranayama), and 3 meditation (dhyana) and relaxation practices. 8 of 10 effective interventions reported the major components of yoga used; all included asana, and 7 also incorporated pranayama and/or dhyana and relaxation practices. Three of the 10 interventions included all of these 3 major components of yoga [55–57, 59, 63], 3 consisted of asana and dhyana and relaxation practices [58, 60, 71], one included asana and pranayama [54], and one included only asana [68]. The majority of these studies were not specific about the exact yoga style, except for 3 which mentioned Hatha yoga [58, 60, 62].

Interventions that included all the 3 major components of yoga reported the time allocated to each component] [55–57, 59, 63]. The median time allocated to asana was around 19 min (IQR 10–25 min), pranayama was around 10 min (IQR 3–32 min), and dhyana and relaxation practice was around 12 min (IQR 10–82 min). Some of the common practices were Tadasana (palm tree pose) [55–57, 59, 60, 68], Virabhadrasana 1&2 (warrior pose) [59, 60, 68], Nadishuddhi pranayama (alternate nostril breathing) [55–57, 63], and Nadanusandhana (A-U-M Kara chanting) [55–57, 63]. Table 4 details the various yoga practices (along with their Sanskrit and English names) effective for pain and/or function [72, 73].

**Table 4:** Yoga practices used in interventions that were effective for pain and/or function

Asana				Pranayama			Dhyana and Relaxation practice		
Sanskrit name	English name	Broad category of yogic pose (standing, sitting, prone, supine)	Effective studies reporting use of the asana for pain and/or function	Sanskrit name	English name	Effective studies reporting use of the pranayama for pain and/or function	Sanskrit name	English name	Effective studies reporting use of the dhyana and relaxation practice for pain and/or function
Parvatasana	Mountain pose	Sitting pose	Pain [54]	Nadishuddhi pranayama	Alternate nostril breathing	Pain [55–57] Function [55–57, 63]	Nadanusandhana	OM meditation/ A,U,M and A-U-M Kara chanting	Pain [55–57] Function [55–57, 63]
Tadasana	Palm tree pose	Standing pose	Pain [55–57, 59, 60, 68] Function [55–57, 60, 68]	Vibhagıya pranayama	Sectional breathing	Function [63]	Savasana	Corpse pose	Function [63]
Ardha kati chakrasana	Half waist wheel pose	Standing pose	Pain [55–57] Function [55–57]	Brahamarı pranayama	Bee breathing	Function [63]	Salamba savasana	Supported corpse pose	Function [71]
Ardha chakrasana	Half wheel pose	Standing pose	Pain [55–57] Function [55–57]	Bhastrika pranayama	Bellows breathing	Function [63]	*NR*	*Cyclic meditation*	*Function *[63]
Prasarita padahastasana	Wide-legged forward bend pose	Standing pose	Pain [55–57, 71] Function [55–57, 71]	Sıtali	Rolling tongue breathing	Function [63]	*NR*	*Mind sound resonance technique*	*Function *[63]
Bhujangasana	Cobra pose	Prone pose	Pain [55–57] Function [55–57, 63]	Sitkarı	Folded tongue breathing	Function [63]	*NR*	*Instant relaxation technique*	*Pain *[55–57] *Function *[55–57, 63]
Shalabasana	Locust pose	Prone pose	Pain [55–57] Function [55–57, 63]	Sadanta	Clenched teeth breathing	Function [63]	*NR*	*Deep relaxation technique*	*Pain *[55–57] *Function *[55–57, 63]
Viparita karani	Legs-up-the-wall pose	Supine pose	Pain [55–57] Function [55–57]	Kapalabhati	Skull shining breathing	Function [63]	*NR*	*Quick relaxation technique*	*Pain*[55–57] *Function*[55–57, 63]
Dhanurasana	Bow pose	Prone pose	Pain [55–57] Function [55–57]	*NR*	*Hands in and out breathing*	*Function *[63]			
Ardha titali asana	Half butterfly	Sitting pose	Pain [55–57] Function [55–57]	*NR*	*Hands stretch breathing*	*Function *[63]			
Titali asana	Full butterfly	Sitting pose	Pain [55–57] Function [55–57]	*NR*	*Ankle stretch breathing*	*Function *[63]			
*NR*	*Hip rotations*	*Standing pose*	*Pain *[55–57] *Function *[55–57]						
Virabhadrasana I	Warrior I pose	Standing pose	Pain [58–60, 68] Function [60, 68]						
Virabhadrasana II	Warrior II pose	Standing pose	Pain [58–60, 68] Function [60, 68]						
Vrksasana	Tree pose	Standing pose	Pain [58, 70] Function [60]						
Utkatasana	Chair pose	Standing pose	Pain [58, 60] Function [60]						
Sukhasana	Easy pose	Sitting pose	Pain [58]						
Baddha konasana	Bound angle pose	Sitting pose	Pain [58, 68] Function [68]						
Upavistha Konasana	Wide-angle seated forward bend pose/straddle seat	Sitting pose	Pain [58, 71]						
Ardha shalabhasana	Half locust pose	Prone pose	Pain [58, 60] Function [60]						
Setu bandha sarvangasana	Bridge pose	Supine pose	Pain [58] Function [63]						
Uttanasana	Standing forward bend	Standing pose	Pain [58, 60] Function [60]						
*NR*	*Hamstring stretches (exact asana NR)*	*Sitting pose*	*Pain*[58]						
Supta baddha konasana	Reclining bound angle pose	Supine pose	Pain [60] Function [60]						
Janu sirsanana	Head-to-knee pose	Sitting pose	Pain [60] Function [60]						
*NR*	*Reclining hamstrings stretch with hip opener with strap*	*Supine pose*	*Pain *[60] *Function *[60]						
Supta matsyendrasana	Reclining twist	Supine pose	Pain [58, 60] Function [60]						
Utthita trikonasana	Extended triangle pose	Standing pose	Pain [68] Function [68]						
Dandasana	Stick pose or staff pose	Sitting pose	Pain [68] Function [68]						
Supta padangustasana	Reclining hand-to-big-toe pose	Supine pose	Pain [68] Function [68]						
Apanasana	Knee to chest pose	Supine pose	Pain [71] Function [71]						
*NR*	*Leg extensions supported squat*	*Standing pose*	*Pain *[71] *Function *[71]						
Paschimottasana	Seated forward bend	Sitting pose	Pain [71] Function [63, 71]						
Bhunamanasana	Earth salutation pose	Sitting pose	Function [63]						
*NR*	*Straddle seat prep standing forward lunge*	*Standing pose*	*Pain *[71] *Function *[71]						
Anantasana/Uttanapadasana	Leg lifts	Supine pose	Pain [71] Function [71]						
*NR*	*Hip lifts*	*Supine pose*	*Pain *[71] *Function *[71]						
*NR*	*Marching one leg balance*	*Sitting/standing pose*	*Pain*[71] *Function*[71]						
*NR*	*Wide leg side bend*	*Standing pose*	*Pain *[71] *Function *[71]						
Skandasana	Standing side lunge	Sitting pose	Pain [71] Function [71]						
Vakrasana	Twisted pose	Sitting pose	Pain [71] Function [71]						
Markatasana	Lumbar stretch pose	Supine pose	Function [63]						

NR: Not reported **Standing pose:** yoga poses practiced with one or both feet on the ground, and the body more or less upright, **Sitting pose:** Yoga poses practiced in seated position, **Supine pose:** Yoga poses practiced in sleeping position, **Prone pose:** Yoga poses performed with the belly or torso touching or facing the floor

The English/Sanskrit names of the yoga practices highlighted in *italics* are the names reported by the authors. It was unclear if these were authentic yoga practices as their standard names were not available from the literature and hence were excluded from the counting for the synthesis of content of yoga interventions

The median duration of centre-based sessions was 8 weeks (interquartile range (IQR) 8–12 weeks) and each session was around 53 min (IQR 45–60 min), and these sessions were mostly delivered once a week (reported in 4 of 8 interventions) [54–58, 60]. Where reported (in 4 of 7 interventions) [55–58, 60, 71], the median duration of home-based sessions was 10 weeks (IQR 8–12 weeks) and each session was around 30 min (IQR 29–30 min), and these sessions were instructed to practice for 4 times a week [58, 60]. One intervention which provided no details on whether it was centre- or home-based, reported session duration and frequency i.e., 30 min thrice a week for 4 weeks [68].

3 of 8 centre-based sessions specifically reported that lectures and counselling on yoga for osteoarthritis, educational materials with written instructions, peer support through group discussion and question-and-answer sessions were used to deliver the intervention [54–57, 63]. To address participants’ needs (e.g., physical limitations), certain yogic poses were adapted in 4 of 10 interventions e.g., by using blocks, straps, blankets, and chairs [58–60, 62]. Where reported (6 of 7 interventions), home-based sessions were delivered by providing written documents with pictures [58–60, 62, 64] and yoga videos (including demonstrations of modified yoga postures to meet individual needs) [71] to the participants.

Strategies were used to monitor and improve adherence to yoga practice at home in 6 of 7 interventions e.g., sharing self-recorded yoga diaries/log sheets, self-recorded yoga videos (documenting home practice details) with the trial team, and reminding and motivating participants e.g., through telephone calls and emails [55–60, 62, 71]. Participants were encouraged to practice yoga in the long term in 2 of 10 interventions [62, 71]*.*

## Discussion

Our review found beneficial effects of yoga on two major symptoms of osteoarthritis i.e., pain and function, as also reported in previous reviews and meta-analyses [28, 29]. However, this review is novel as it aims to identify and synthesise the key features (content, structure, and delivery characteristics) of effective yoga interventions. Although the components of effective yoga interventions were heterogeneous, some commonalities were identified. A majority of them involved participants attending yoga at a centre for supervised group sessions, once a week for a sustained period. Most also involved individual yoga practice at home, which was unsupervised, several times a week between centre-based sessions. These interventions generally incorporated yoga postures (asana), but a majority also included other major components including breathing practices (pranayama) and/or meditation (dhyana) and relaxation practices. Keeping in mind the participants’ needs (e.g., knee/hip pain), various modifications were provided to certain yogic poses using props (e.g., a chair as a support for standing poses). The review also indicated ways in which these interventions ensured maximum adherence to yoga among participants, especially to yoga practice at home, through regular contact with the participant or journaling of home practice.

Yoga has been recommended by an international osteoarthritis evidence-based clinical guideline developed by the American College of Rheumatology (ACR) and the Arthritis Foundation (AF) [10]. This guideline used the GRADE methodology to rate the quality of the available evidence and to develop the recommendations [10]. The guideline was developed by an expert panel representing the ACR, including rheumatologists, an internist, physical and occupational therapists, and osteoarthritis patients. The guideline “conditionally” recommends the use of yoga for managing symptoms of knee osteoarthritis. The conditional recommendation inferred that yoga could be used by the patients to manage knee osteoarthritis only after shared decision-making, including a detailed explanation of the benefits and harms of yoga to the patients, in a language and context they understand. However, due to a lack of evidence, no recommendations could be made for managing hand and hip osteoarthritis through yoga [10]. It is important to note that this guideline was based on a generic definition of yoga laid out by the National Center for Complementary and Integrative Health [NCCIH] as “a mind–body practice consisting of physical postures, breathing techniques, and meditation or relaxation”. Yoga includes a diverse range of components, so it is important to consider the aspects of yoga that are effective.

To the best of our knowledge, this is the first systematic review conducted by following a robust methodology to synthesise the content, structure, and delivery characteristics of effective yoga interventions for managing major osteoarthritis symptoms. Determination of the effectiveness of interventions was standardised across studies by using meta-analysis before finally the detailed characteristics of potentially effective yoga interventions were synthesised. Although we used a comprehensive search, only 16 studies met our inclusion criteria, 2 of which could not be included in the meta-analysis.

Our review highlighted some limitations in the included studies. Some studies had incomplete or unclear information on the time allocated to each component of the yoga intervention. Future RCTs should improve the reporting of this detail which would help in establishing the aspects of yoga practice that are most beneficial for improving osteoarthritis symptoms and therefore where the emphasis for future yoga-based interventions should lie. Most studies did not describe the qualifications of the yoga providers, instead only described the yoga providers as certified, experienced, or trained. Where yoga provider qualifications were reported in detail, there was no description of the yoga providers' training to deliver the specific intervention protocol. The expertise of yoga providers and their training in the intervention protocol are likely to be essential to the effectiveness of the intervention and in ensuring the fidelity and safety of the intervention [74, 75]. Thus, future RCTs should ensure adequate reporting of the training details of the yoga providers. In terms of safety, a majority of the studies did not explicitly report adverse events related to yoga. This issue should be addressed and reporting should be improved in future RCTs, to indicate their safety. Most included studies were based in the US, predominantly included females, and reported on knee osteoarthritis. These factors potentially limit the generalisability of our findings. Existing evidence has shown a higher uptake of yoga among women as compared to men [11]. Although osteoarthritis is more common in women, it also affects men. So, it is important to explore the possible barriers to yoga practice among men (e.g., gender-based perceptions and their preference for other forms of physical activity) and take some initiatives (e.g., men-only yoga classes) to promote yoga among men for osteoarthritis, in future RCTs [76]. Lastly, most included studies involved relatively short-term follow-up, and only 2 of our included studies reported on encouraging participants to continue yoga practice. For a chronic condition like osteoarthritis, it will be important to explore whether yoga practice and its effects are sustained in the longer term.

Despite the low quality and heterogeneity of included studies, our findings suggest yoga interventions might be effective in managing osteoarthritis symptoms and highlight the key characteristics of effective yoga interventions for osteoarthritis. Supervised yoga practice in groups at a centre seemed to be one of the most consistent characteristics in the studies included in this review. This might reflect the importance of creating a community for yoga practice, given that most people with osteoarthritis are older, and may be socially isolated [77]. Older participants generally tend to be interested and adherent to yoga classes (at a centre) [78], which may also be why centre-based yoga sessions are an important feature of yoga practice. Adherence of participants to the yoga interventions is likely to play an important role in its effectiveness on osteoarthritis symptoms [79]. Most studies included in this review mentioned strategies such as self-reporting of home practice and ensuring regular contact with the participants to encourage them to adhere to yoga practice at home, though the level of adherence was generally not reported. To improve adherence to mind–body interventions such as yoga, some strategies including providing reminders, following up with the individual to ensure regular yoga practice, and catering to individual needs (e.g., modifying yogic poses), have been identified [80]. Further, it will be important to explore how yoga interventions might be implemented and integrated within the orthopaedic healthcare system [81]. Hence, future research should aim to develop a yoga program for managing osteoarthritis by using the synthesised findings from this review. This is likely to require the input of a variety of stakeholders, including osteoarthritis patients, yoga providers, and orthopaedic doctors to address the potential challenges and to consider the evidence base for its effective and safe incorporation, by using Delphi or similar methods.

## Conclusion

Considering the methodological limitations of previous studies, including low quality of studies and heterogeneity between studies, a high-quality long-term RCT should be conducted to determine the effectiveness of yoga in managing osteoarthritis symptoms by using the synthesised key characteristics of previous effective yoga interventions, as identified by this review.

### Supplementary Information

Below is the link to the electronic supplementary material.

Supplementary file1 (DOCX 48 KB)

## References

[1] National Health Service (2023) Osteoarthritis. https://www.nhs.uk/conditions/osteoarthritis/. Accessed 20 July 2023

[2] GBD (2019) Global burden of 369 diseases and injuries in 204 countries and territories, 1990–2019: a systematic analysis for the Global Burden of Disease Study 2019. https://vizhub.healthdata.org/gbd-results/ Accessed 20 July 2023 10.1016/S0140-6736(20)30925-9 PMC7567026 33069326

[3] Sharma A, Kudesia P, Shi Q, Gandhi R (2016) Anxiety and depression in patients with osteoarthritis: impact and management challenges. Open Access Rheumatol Res Rev 8:103–113. 10.2147/OARRR.S93516 10.2147/OARRR.S93516 PMC5098683 27843376

[4] Hunter DJ, Schofield D, Callander E (2014) The individual and socioeconomic impact of osteoarthritis. Nat Rev Rheumatol 10:437–441. 10.1038/nrrheum.2014.44 24662640 10.1038/nrrheum.2014.44

[5] Leifer VP, Katz JN, Losina E (2022) The burden of OA-health services and economics. Osteoarthr Cartil 30:10–16. 10.1016/j.joca.2021.05.007 10.1016/j.joca.2021.05.007 PMC8605034 34023527

[6] Hunter DJ, March L, Chew M (2020) Osteoarthritis in 2020 and beyond: a Lancet commission. Lancet 36:10264. 10.1016/s0140-6736(20)32230-3 10.1016/s0140-6736(20)32230-3 33159851

[7] National Institute for Health and Care Excellence (NICE) (2020) Osteoarthritis: care and management. https://www.ncbi.nlm.nih.gov/books/NBK568417/ Accessed 25 November 2023

[8] Buelt A, Narducci DM (2021) Osteoarthritis management: updated guidelines from the American College of Rheumatology and Arthritis Foundation. Am Fam Physician 103(2):120–121 33448759

[9] Marks R (2012) Knee osteoarthritis and exercise adherence: a review. Curr aging Sci 5(1):72–83 21762086 10.2174/1874609811205010072

[10] Kolasinski SL, Neogi T, Hochberg MC et al (2020) 2019 American College of Rheumatology/Arthritis Foundation Guideline for the management of osteoarthritis of the hand, hip, and knee. ArthritisRheumatol 72(2):220–233. 10.1002/art.41142 10.1002/art.41142 PMC10518852 31908163

[11] Cartwright T, Mason H, Porter A, Pilkington K (2020) Yoga practice in the UK: a cross-sectional survey of motivation, health benefits and behaviours. BMJ Open 10(1):e031848. 10.1136/bmjopen-2019-031848 31932388 10.1136/bmjopen-2019-031848 PMC7044896

[12] Cramer H, Ward L, Steel A, Lauche R, Dobos G, Zhang Y (2016) Prevalence, patterns, and predictors of yoga use: results of a U.S. nationally representative survey. Am J Prev Med 50:230–235. 10.1016/j.amepre.2015.07.037 26497261 10.1016/j.amepre.2015.07.037

[13] Desikachar K, Bragdon L, Bossart C (2005) The yoga of healing: exploring yoga’s holistic model for health and well-being. Int J Yoga Therap 15:17–39. 10.17761/IJYT.15.1.P501L33535230737 10.17761/IJYT.15.1.P501L33535230737

[14] Lasater J (1997) The heart of Patanjali. Yoga J 137:134–144

[15] McCall T (2007) *Yoga as medicine: the yogic prescription for health and healing.* New York, NY

[16] Fishman L, Saltonstall E (2008) *Yoga for arthritis*. New York, NY

[17] Telles S, Singh N (2013) Science of the mind: ancient yoga texts and modern studies. Psychiatr Clin N Am 36(1):93–108. 10.1016/j.psc.2013.01.010 10.1016/j.psc.2013.01.010 23538080

[18] Goncalves LC, Vale RG, Barata NJ, Varejao RV, Dantas EHM (2011) Flexibility, functional autonomy and quality of life (QoL) in elderly yoga practitioners. Arch Gerontol Geriatr 53(2):158–162. 10.1016/j.archger.2010.10.028 21167613 10.1016/j.archger.2010.10.028

[19] Rathore M, Trivedi S, Abraham J, Sinha MB (2017) Anatomical correlation of core muscle activation in different yogic postures. Int J Yoga 10(2):59–66. 10.4103/0973-6131.205515 28546675 10.4103/0973-6131.205515 PMC5433114

[20] Zuckerman A (2020) Significant yoga statistics: 2020/2021 benefits, facts and trends. https://comparecamp.com/yoga-statistics/#TOC2 Accessed 20 November 2023

[21] Anderson JG, Taylor AG (2011) The metabolic syndrome and mind-body therapies: a systematic review. J Nutr Metab. 10.1155/2011/276419 21773016 10.1155/2011/276419 PMC3136147

[22] National Health Service (NHS) (2018) Exercise. https://www.nhs.uk/live-well/exercise/ Accessed 30 November 2023

[23] Kocyigit BF, Sagtaganov Z, Yessirkepov M (2023) The effectiveness of yoga as a form of exercise in the management of rheumatic diseases. Rheumatol Int 43(5):795–801. 10.1007/s00296-023-05291-9 36856817 10.1007/s00296-023-05291-9

[24] Sharma M (2014) Yoga as an alternative and complementary approach for arthritis: a systematic review. J Evid Based Complement Altern Med 19(1):51–58. 10.1177/2156587213499918 10.1177/2156587213499918 24647379

[25] Ward L, Stebbings S, Cherkin D, Baxter GD (2014) Components and reporting of yoga interventions for musculoskeletal conditions: a systematic review of randomised controlled trials. Complement Ther Med 22(5):909–919. 10.1016/j.ctim.2014.08.007 25440383 10.1016/j.ctim.2014.08.007

[26] Kan L, Zhang J, Yang Y, Wang P (2016) The effects of yoga on pain, mobility, and quality of life in patients with knee osteoarthritis: a systematic review. Evid Based Complement Altern Med. 10.1155/2016/6016532 10.1155/2016/6016532 PMC5061981 27777597

[27] Wang Y, Lu S, Wang R et al (2018) Integrative effect of yoga practice in patients with knee arthritis: a PRISMA-compliant meta-analysis. Medicine 97(31):e11742. 10.1097/md.0000000000011742 30075589 10.1097/md.0000000000011742 PMC6081169

[28] Lauche R, Hunter DJ, Adams J, Cramer H (2019) Yoga for osteoarthritis: a systematic review and meta-analysis. Curr Rheumatol Rep. 10.1007/s11926-019-0846-5 31338685 10.1007/s11926-019-0846-5

[29] Zampogna B, Papalia R, Papalia GF et al (2020) The role of physical activity as a conservative treatment for hip and knee osteoarthritis in older people: a systematic review and meta-analysis. J Clin Med. 10.3390/jcm9041167 32325775 10.3390/jcm9041167 PMC7230847

[30] Denham-Jones L, Gaskell L, Spence N, Pigott T (2022) A systematic review of the effectiveness of yoga on pain, physical function, and quality of life in older adults with chronic musculoskeletal conditions. Musculoskeletal Care 20(1):47–73. 10.1002/msc.1576 34125986 10.1002/msc.1576

[31] Tufanaru C, Munn Z, Aromataris E, et al (2017) Chapter 3: Systematic reviews of effectiveness. The Joanna Briggs Institute: Adelaide, Australia. http://reviewersmanual.joannabriggs.org Accessed 20 November 2023

[32] Page MJ, McKenzie JE, Bossuyt PM et al (2021) The PRISMA 2020 statement: an updated guideline for reporting systematic reviews. PLoS Med 18(3):e1003583. 10.1371/journal.pmed.1003583 33780438 10.1371/journal.pmed.1003583 PMC8007028

[33] Biswas I, Lewis S, Chattopadhyay K (2022) Content, structure and delivery characteristics of yoga interventions for the management of osteoarthritis: a systematic review protocol. Int J Environ Res Public Health. 10.3390/ijerph19105806 35627341 10.3390/ijerph19105806 PMC9140376

[34] Fernandes L, Hagen KB, Bijlsma JWJ et al (2013) EULAR recommendations for the non-pharmacological core management of hip and knee osteoarthritis. Ann Rheum Dis 72(7):1125–1135. 10.1136/annrheumdis-2012-202745 23595142 10.1136/annrheumdis-2012-202745

[35] National Institute for Health and Care Excellence (NICE) (2015) Osteoarthritis in over 16s. https://www.nice.org.uk/guidance/qs87 Accessed 26 November 2023

[36] Rolfson O, Wissig S, van Maasakkers L et al (2016) Defining an international standard set of outcome measures for patients with hip or knee osteoarthritis: consensus of the international consortium for health outcomes measurement hip and knee osteoarthritis working group. Arthritis Care Res 68(11):1631–1639. 10.1002/acr.22868 10.1002/acr.22868 PMC5129496 26881821

[37] American Academy of Orthopaedic Surgeons (2017) Osteoarthritis: Function and Pain Assessment Measure Methodology Report. https://osteoarthritis-function-and-pain-assessment-final-report-approved-by-b.pdf (aaos.org) Accessed 26 November 2023.

[38] European Medicines Agency (2010) Guideline on clinical investigation of medicinal products used in the treatment of osteoarthritis. https://www.ema.europa.eu/en/clinical-investigation-medicinal-products-used-treatment-osteoarthritis-scientific-guideline Accessed 28 November 2023.

[39] Nalbant G, Hassanein ZM, Lewis S, Chattopadhyay K (2022) Content, structure and delivery characteristics of yoga interventions for managing hypertension: a systematic review and meta-analysis of randomised controlled trials. Front Public Health. 10.3389/fpubh.2022.846231 35419342 10.3389/fpubh.2022.846231 PMC8995771

[40] National Institute for Health and Care Excellence (2022) Osteoarthritis: assessment and management NICE guideline: search strategies. https://www.nice.org.uk/guidance/ng226/documents/search-strategies Accessed 28 November 2023.

[41] Cameron M, Chrubasik S (2014) Oral herbal therapies for treating osteoarthritis. Cochrane Database Syst Rev. 10.1002/14651858.cd002947.pub2 24848732 10.1002/14651858.cd002947.pub2 PMC4494689

[42] Fransen M, McConnell S, Hernandez-Molina G, Reichenbach S (2014) Exercise for osteoarthritis of the hip. Cochrane Database Syst Rev. 10.1002/14651858.cd007912.pub2 24756895 10.1002/14651858.cd007912.pub2 PMC10898220

[43] Higgins JPT, Thomas J, Chandler J, et al (2021) Cochrane handbook for systematic reviews of interventions Version 6.2. https://training.cochrane.org/handbook/current Accessed 03 December 2023.

[44] Cochrane training (2023) RCT Filter used by Cochrane ENT. https://training.cochrane.org/handbook/current/chapter-04#section-4-4-7 Accessed 03 December 2023.

[45] ISSG Search Filter Resource. https://sites.google.com/a/york.ac.uk/issg-search-filters-resource/home/rcts?authuser=0 Accessed 03 December 2023.

[46] Endnote X9 Clarivate Analytics (2019) Endnote. http://endnote.com/ Accessed 05 December 2023.

[47] Mourad O, Hossam H, Zbys F, Ahmed E (2016) Rayyan—a web and mobile app for systematic reviews. Syst Rev 5(1):210. 10.1186/s13643-016-0384-4 27919275 10.1186/s13643-016-0384-4 PMC5139140

[48] James KA, Heideken JV, Iversen MD (2021) Reporting of adverse events in randomized controlled trials of therapeutic exercise for hip osteoarthritis: a systematic review. Phys Ther. 10.1093/ptj/pzab195 34730830 10.1093/ptj/pzab195 PMC8565302

[49] Moher D, Hopewell S, Schulz KF et al (2010) CONSORT 2010 explanation and elaboration: updated guidelines for reporting parallel group randomised trials. Int J Surg 10(1):28–55. 10.1016/j.ijsu.2011.10.001 10.1016/j.ijsu.2011.10.001 22036893

[50] Beckett RD, Loeser KC, Bowman KR, Towne TG (2016) Intention-to-treat and transparency of related practices in randomized, controlled trials of anti-infectives. BMC Med Res Methodol. 10.1186/s12874-016-0215-2 27557676 10.1186/s12874-016-0215-2 PMC4997732

[51] Vickers AJ (2001) The use of percentage change from baseline as an outcome in a controlled trial is statistically inefficient: a simulation study. BMC Med Res Methodol. 10.1186/1471-2288-1-6 11459516 10.1186/1471-2288-1-6 PMC34605

[52] Higgins JPT, Li T, Deeks JJ (2019) Chapter 6: Choosing effect measures and computing estimates of effect. In Cochrane Handbook for Systematic Reviews of Interventions; Version 6.0; Higgins JPT, Thomas J, Chandler J, Cumpston M, Li, T, Page MJ. Eds.; Welch: Cochrane, VA, Canada.

[53] Review Manager (RevMan) (2020) The Cochrane Collaboration.

[54] Garfinkel MS, Schumacher HR Jr, Husain A, Levy M, Reshetar RA (1994) Evaluation of a yoga-based regimen for treatment of osteoarthritis of the hands. J Rheumatol 21(12):2341–2343 7699639

[55] Ebnezar J, Nagarathna R, Bali Y, Nagendra HR (2011) Effect of an integrated approach of yoga therapy on quality of life in osteoarthritis of the knee joint: a randomized control study. Int J Yoga 4(2):55–63. 10.4103/0973-6131.85486 22022123 10.4103/0973-6131.85486 PMC3193655

[56] Ebnezar J, Nagarathna R, Yogitha B, Nagendra HR (2012) Effect of integrated yoga therapy on pain, morning stiffness and anxiety in osteoarthritis of the knee joint: a randomized control study. Int J Yoga 5(1):28–36. 10.4103/0973-6131.91708 22346063 10.4103/0973-6131.91708 PMC3276929

[57] Ebnezar J, Nagarathna R, Bali Y, Nagendra HR (2012) Effects of an integrated approach of Hatha yoga therapy on functional disability, pain, and flexibility in osteoarthritis of the knee joint: a randomized controlled study. J Altern Complement Med 18(5):463–472. 10.1089/acm.2010.0320 22537508 10.1089/acm.2010.0320

[58] Cheung C, Wyman JF, Resnick B, Savick K (2014) Yoga for managing knee osteoarthritis in older women: a pilot randomized controlled trial. BMC Complement Altern Med. 10.1186/1472-6882-14-160 24886638 10.1186/1472-6882-14-160 PMC4038088

[59] Park J, Newman D, McCaffrey R, Garrido JJ, Riccio ML, Liehr P (2016) The effect of chair yoga on biopsychosocial changes in English- and Spanish-speaking community-dwelling older adults with lower-extremity osteoarthritis. J Gerontol Soc Work 59(7–8):604–626. 10.1080/01634372.2016.1239234 27661469 10.1080/01634372.2016.1239234 PMC5177482

[60] Cheung C, Wyman JF, Bronas U, McCarthy T, Rudser K, Mathiason MA (2017) Managing knee osteoarthritis with yoga or aerobic/strengthening exercise programs in older adults: a pilot randomized controlled trial. Rheumatol Int 37(3):389–398. 10.1007/s00296-016-3620-2 27913870 10.1007/s00296-016-3620-2 PMC5569242

[61] McCaffrey R, Park J, Newman D (2017) Chair Yoga: feasibility and sustainability study with older community-dwelling adults with osteoarthritis. Holist Nurs Pract 31(3):148–157. 10.1097/hnp.0000000000000184 27782922 10.1097/hnp.0000000000000184

[62] Park J, McCaffrey R, Newman D, Liehr P, Ouslander JG (2017) A pilot randomized controlled trial of the effects of chair yoga on pain and physical function among community-dwelling older adults with lower extremity osteoarthritis. J Am Geriatr Soc 65(3):592–597. 10.1111/jgs.14717 28008603 10.1111/jgs.14717 PMC5357158

[63] Deepeshwar S, Tanwar M, Kavuri V, Budhi RB (2018) Effect of yoga-based lifestyle intervention on patients with knee osteoarthritis: a randomized controlled trial. Front Psychiatry. 10.3389/fpsyt.2018.00180 29867604 10.3389/fpsyt.2018.00180 PMC5952125

[64] Kaur R (2018) Determinants of impact of two intervention packages on quality of life of 30–60-year-old women suffering from knee osteoarthritis in Gurdaspur Punjab a community-based trial. Dissertation, Panjab University

[65] Kuntz A, Chopp Hurley JN, Brenneman EC et al (2018) Efficacy of a biomechanically based yoga exercise program in knee osteoarthritis: a randomized controlled trial. PLoS ONE. 10.1371/journal.pone.0195653 29664955 10.1371/journal.pone.0195653 PMC5903657

[66] Zacharia S, Taylor EL, Branscum PW, Cheney M, Hofford CW, Crowson M (2018) Effects of a yoga intervention on adults with lower limb osteoarthritis: a randomized controlled trial. Am J Health Studies. 10.47779/ajhs.2018.60 10.47779/ajhs.2018.60

[67] McCaffrey R, Taylor D, Marker C, Park J (2019) A pilot study of the effects of chair yoga and chair-based exercise on biopsychosocial outcomes in older adults with lower extremity osteoarthritis. Holist Nurs Pract 33(6):321–326. 10.1097/hnp.0000000000000355 31609869 10.1097/hnp.0000000000000355

[68] Vaghela N, Mishra D, Patel J, Dani V (2020) Promoting health and quality of life of patients with osteoarthritis of knee joint through non-pharmacological treatment strategies: a randomized controlled trial. J Educ Health Promot. 10.4103/jehp.jehp_39_20 32766341 10.4103/jehp.jehp_39_20 PMC7377148

[69] Bokaeian HR, Esfandiarpour F, Zahednejad S, Mohammadi HK, Farahmand F (2021) Effects of an exercise therapy targeting knee kinetics on pain, function, and gait kinetics in patients with knee osteoarthritis: a randomized clinical trial. Adapt Phys Activ Q 38(3):377–395. 10.1123/apaq.2020-0144 33785660 10.1123/apaq.2020-0144

[70] Park J, Herron C (2021) Effects of a movement-based mind-body intervention in managing osteoarthritis symptoms in older adults. Innov Aging. 10.1093/geroni/igab046.1767 10.1093/geroni/igab046.1767

[71] Bennell KL, Schwartz S, Teo PL et al (2022) Effectiveness of an unsupervised online yoga program on pain and function in people with knee osteoarthritis: a randomized clinical trial. Ann Intern Med 175(10):1345–1355. 10.7326/m22-1761 36122378 10.7326/m22-1761

[72] Chattopadhyay K, Mishra P, Manjunath NK et al (2020) Development of a yoga program for Type-2 Diabetes Prevention (YOGA-DP) among high-risk people in India. Front Public Health. 10.3389/fpubh.2020.548674 33313032 10.3389/fpubh.2020.548674 PMC7706999

[73] Yoga Journal (2023) A-Z directory of yoga poses. https://www.yogajournal.com/pose-finder/pose-finder/ Accessed 05 May 2023.

[74] Hoffmann TC, Glasziou PP, Boutron I et al (2014) Better reporting of interventions: template for intervention description and replication (TIDieR) checklist and guide. BMJ. 10.1136/bmj.g1687 24609605 10.1136/bmj.g1687

[75] Ward L, Nault D, Cramer H, Moonaz S (2022) Development of the CLARIFY (CheckList stAndardising the Reporting of Interventions For Yoga) guidelines: a Delphi study. BMJ Open. 10.1136/bmjopen-2021-054585 35105638 10.1136/bmjopen-2021-054585 PMC8804643

[76] Cagas JY, Biddle SJH, Vergeer I (2021) Yoga not a (physical) culture for men? Understanding the barriers for yoga participation among men. Complement Ther Clin Pract. 10.1016/j.ctcp.2020.101262 33276223 10.1016/j.ctcp.2020.101262

[77] Siviero P, Veronese N, Smith T et al (2020) Association between osteoarthritis and social isolation: data from the EPOSA Study. J Am Geriatr Soc 68(1):87–95. 10.1111/jgs.16159 31529624 10.1111/jgs.16159 PMC6954097

[78] Flegal KE, Kishiyama S, Zajdel D, Haas M, Oken BS (2007) Adherence to yoga and exercise interventions in a 6-month clinical trial. BMC Complement Altern Med. 10.1186/1472-6882-7-37 17996075 10.1186/1472-6882-7-37 PMC2194735

[79] Cheung C, Wyman JF, Savik K (2016) Adherence to a yoga program in older women with knee osteoarthritis. J Aging Phys Act 24(2):181–188. 10.1123/japa.2015-0048 26214142 10.1123/japa.2015-0048

[80] Salmon PG, Santorelli SF, Kabat-Zinn J (1998) Intervention elements promoting adherence to mindfulness-based stress reduction programs in the clinical behavioral medicine setting. In: Shumaker SA, Schron EB, Ockene JK, McBee WL (ed) The Handbook of Health Behavior Change. New York: Springer Publishing Co, New York, pp 239–266

[81] Mason H, Schnackenberg N, Monro R (2017) Yoga and healthcare in the United Kingdom. Int J Yoga Therap 27(1): 121–126. 10.17761/1531-2054-27.1.121 10.17761/1531-2054-27.1.121 29131732

