# Supplementary material for: Key characteristics of effective yoga interventions for managing osteoarthritis: a systematic review and meta-analysis
Source: Rheumatol Int. 2024 Jun 27;44(9):1647–77. doi: 10.1007/s00296-024-05652-y (PMC11343886; doi:10.1007/s00296-024-05652-y)
Supplement: Supplementary file 1 — Supplementary file1 (DOCX 48 KB) [file 296_2024_5652_MOESM1_ESM.docx]

Appendix 1: Search strategies

**1. MEDLINE (Ovid) <1946 to 22 September 2023>: 210 records**

1. exp Mind-Body Therapies/

2. mind body therap*.mp.

3. meditat*.mp.

4. (yoga* or yogi*).mp.

5. (Asana* or pranayam* or dhyan* or ashtanga or bikram or hatha or iyengar or kripalu or kundalini or vinyasa or raja or radja or bhakti or jnana or kriya* or karma or yama or niyama or pratyahara or dharana or samadhi or bandha or mudra* or chanda or sivananda).mp.

6. 1 or 2 or 3 or 4 or 5

7. exp Osteoarthritis/

8. (osteoarthr* or osteo-arthr*).mp.

9. Coxarthrosis.mp.

10. Arthrosis deformans.mp.

11. (Degenerative adj2 arthritis).mp.

12. Degenerative joint disease*.mp.

13. Non-inflammatory arthritis.mp.

14. 7 or 8 or 9 or 10 or 11 or 12 or 13

15. randomized controlled trial.pt.

16. controlled clinical trial.pt.

17. randomized.ab.

18. placebo.ab.

19. drug therapy.fs.

20. randomly.ab.

21. trial.ab.

22. groups.ab.

23. 15 or 16 or 17 or 18 or 19 or 20 or 21 or 22

24. exp animals/ not humans.sh.

25. 23 not 24

26. 6 and 14 and 25

**2. EMBASE (Ovid) <1974 to 22 September 2023>: 550 records**

1 exp alternative medicine/

2 exp yoga/

3 exp meditation/

4 Mind body therap*.mp.

5 (Yoga* or yogi*).mp.

6 Meditat*.mp.

7 (Asana* or pranayam* or dhyan* or ashtanga or bikram or hatha or iyengar or kripalu or kundalini or vinyasa or raja or radja or bhakti or jnana or kriya* or karma or yama or niyama or pratyahara or dharana or samadhi or bandha or mudra* or chanda or sivananda).mp.

8 1 or 2 or 3 or 4 or 5 or 6 or 7

9 exp osteoarthritis/

10 (osteoarthr* or osteo-arthr*).mp.

11 Coxarthrosis.mp.

12 Arthrosis deformans.mp.

13 (Degenerative adj2 arthritis).mp.

14 Degenerative joint disease*.mp.

15 Non-inflammatory arthritis.mp.

16 9 or 10 or 11 or 12 or 13 or 14 or 15

17 Randomized controlled trial/

18 Controlled clinical trial/

19 random*.ti,ab.

20 randomization/

21 intermethod comparison/

22 placebo.ti,ab.

23 (compare or compared or comparison).ti.

24 ((evaluated or evaluate or evaluating or assessed or assess) and (compare or compared or comparing or comparison)).ab.

25 (open adj label).ti,ab.

26 ((double or single or doubly or singly) adj (blind or blinded or blindly)).ti,ab.

27 double blind procedure/

28 parallel group*1.ti,ab.

29 (crossover or cross over).ti,ab.

30 ((assign* or match or matched or allocation) adj5 (alternate or group*1 or intervention*1 or patient*1 or subject*1 or participant*1)).ti,ab.

31 (assigned or allocated).ti,ab.

32 (controlled adj7 (study or design or trial)).ti,ab.

33 (volunteer or volunteers).ti,ab.

34 human experiment/

35 trial.ti.

36 17 or 18 or 19 or 20 or 21 or 22 or 23 or 24 or 25 or 26 or 27 or 28 or 29 or 30 or 31 or 32 or 33 or 34 or 35.

37 (random* adj sampl* adj7 (cross section* or questionnaire*1 or survey* or database*1)).ti,ab. not (comparative study/ or controlled study/ or randomi?ed controlled.ti,ab. or randomly assigned.ti,ab.)

38 Cross-sectional study/ not (randomized controlled trial/ or controlled clinical study/ or controlled study/ or randomi?ed controlled.ti,ab. or control group*1.ti,ab.)

39 (((case adj control*) and random*) not randomi?ed controlled).ti,ab.

40 (Systematic review not (trial or study)).ti.

41 (nonrandom* not random*).ti,ab.

42 Random field*.ti,ab.

43 (random cluster adj3 sampl*).ti,ab.

44 (review.ab. and review.pt.) not trial.ti.

45 we searched.ab. and (review.ti. or review.pt.)

46 update review.ab.

47 (databases adj4 searched).ab.

48 (rat or rats or mouse or mice or swine or porcine or murine or sheep or lambs or pigs or piglets or rabbit or rabbits or cat or cats or dog or dogs or cattle or bovine or monkey or monkeys or trout or marmoset$1).ti. and animal experiment/

49 Animal experiment/ not (human experiment/ or human/)

50 37 or 38 or 39 or 40 or 41 or 42 or 43 or 44 or 45 or 46 or 47 or 48 or 49

51 36 not 50

52 8 and 16 and 51

**3. PsycInfo (OVID) <1806 to 22 September 2023>: 14 records**

1 exp Mind Body Therapy/

2 Mind-body therap*.mp.

3 exp Yoga/

4 (Yoga* or yogi*).mp.

5 exp Meditation/

6 Meditat*.mp.

7 (Asana* or pranayam* or dhyan* or ashtanga or bikram or hatha or iyengar or kripalu or kundalini or vinyasa or raja or radja or bhakti or jnana or kriya* or karma or yama or niyama or pratyahara or dharana or samadhi or bandha or mudra* or chanda or sivananda).mp.

8 1 or 2 or 3 or 4 or 5 or 6 or 7

9 (Osteoarthr* or osteo-arthr*).mp.

10 Coxarthrosis.mp.

11 Arthrosis deformans.mp.

12 (Degenerative adj2 arthritis).mp.

13 Degenerative joint disease.mp.

14 Non-inflammatory arthritis.mp.

15 9 or 10 or 11 or 12 or 13 or 14

16 (Randomized Controlled Trial or Controlled Clinical Trial or Pragmatic Clinical Trial or Equivalence Trial or Clinical Trial, Phase III).pt.

17 Randomized Controlled Trial/

18 exp Randomized Controlled Trials/

19 “Randomized Controlled Trial.mp

20 Controlled Clinical Trial/

21 Controlled Clinical Trials/

22 exp Clinical Trials/

23 “Controlled Clinical Trial.mp

24 Randomization/

25 Random Allocation/

26 Double-Blind Method/

27 Double Blind Procedure/

28 Double-Blind Studies/

29 Single-Blind Method/

30 Single Blind Procedure/

31 Single-Blind Studies/

32 Placebos/

33 Placebo/

34 Control Groups/

35 Control Group/

36 (random* or sham or placebo*).ti,ab,hw.

37 ((singl* or doubl*) adj (blind* or dumm* or mask*)).ti,ab,hw.

38 ((tripl* or trebl*) adj (blind* or dumm* or mask*)).ti,ab,hw.

39 (control* adj3 (study or studies or trial* or group*)).ti,ab.

40 (Nonrandom* or non random* or non-random* or quasi-random* or quasirandom*).ti,ab,hw.

41 allocated.ti,ab,hw.

42 ((open label or open-label) adj5 (study or studies or trial*)).ti,ab,hw.

43 ((equivalence or superiority or non-inferiority or noninferiority) adj3 (study or studies or trial*)).ti,ab,hw.

44 (pragmatic study or pragmatic studies).ti,ab,hw.

45 ((pragmatic or practical) adj3 trial*).ti,ab,hw.

46 ((quasiexperimental or quasi-experimental) adj3 (study or studies or trial*)).ti,ab,hw.

47 (phase adj3 III adj3 (study or studies or trial*)).ti,hw.

48 16 or 17 or 18 or 19 or 20 or 21 or 22 or 23 or 24 or 25 or 26 or 27 or 28 or 29 or 30 or 31 or 32 or 33 or 34 or 35 or 36 or 37 or 38 or 39 or 40 or 41 or 42 or 43 or 44 or 45 or 46 or 47

49 8 and 15 and 48

**4. CINAHL (EBSCOHost)** **<1994 to 22 September 2023>: 539 records**

S1 (MH “Yoga”)

S2 (MH “Mind Body Techniques+”)

S3 (MH “Meditation”)

S4 TX Yoga* or yogi*

S5 TX “Mind body therap*”

S6 TX Meditat*

S7 TX pranayam* or dhyan* or ashtanga or bikram or hatha or iyengar or kripalu or kundalini or vinyasa or raja or radja or bhakti or jnana or kriya* or karma or yama or niyama or pratyahara or dharana or samadhi or bandha or mudra* or chanda or sivananda

S8 S1 or S2 or S3 or S5 or S6 or S7

S9 MH Osteoarthritis

S10 TX Osteoarthr* or Osteo-arthr*

S11 TX Arthrosis deformans

S12 TX Coxarthrosis

S13 TX Degenerative adj2 arthritis

S14 TX Degenerative arthritis

S15 TX Degenerative joint disease*

S16 TX Non-inflammatory arthritis

S17 S9 or S10 or S11 or S12 or S13 or S14 or S15 or S16

S18 (MH “Randomized Controlled Trials”)

S19 (MH “Double-Blind Studies”)

S20 (MH “Single-Blind Studies”)

S21 (MH “Random Assignment”)

S22 (MH “Pretest-Posttest Design”)

S23 (MH “Cluster Sample”)

S24 TI (randomised or randomized)

S25 AB (random*)

S26 TI (trial)

S27 TI (sample size) and AB (assigned OR allocated OR control)

S28 MH placebos

S29 PT randomized controlled trial

S30 AB control W5 group

S31 AB (crossover design) or MH (comparative studies)

S32 AB cluster W3 RCT

S33 MH animals+

S34 MH animal studies

S35 TI animal model*

S36 S33 or S34 or S35

S37 MH human

S38 S36 not S37

S39 S18 or S19 or S20 or S21 or S22 or S23 or S24 or S25 or S26 or S27 or S28 or S29 or S30 or S31 or S32

S40 S39 not S38

S41 S8 and S17 and S40

**5. Cochrane Central Register of Controlled Trials (CENTRAL) <1996 to 22 September 2023>: 266 records**

#1 MeSH descriptor: [Mind-Body Therapies] explode all trees

#2 MeSH descriptor: [Meditation] explode all trees

#3 (yoga*) (Word variations have been searched)

#4 (Mind body therapies) (Word variations have been searched)

#5 (yogi*) (Word variations have been searched)

#6 (asana* or pranayam* or dhyan* or meditat* or ashtanga or bikram or hatha or iyengar or kripalu or kundalini or vinyasa or raja or radja or bhakti or jnana or kriya* or karma or yama or niyama or pratyahara or dharana or samadhi or bandha or mudra* or chanda or sivananda) (Word variations have been searched)

#7 #1 or #2 or #3 or #4 or #5 or #6

#8 MeSH descriptor: [Osteoarthritis] explode all trees

#9 (osteoarthr*) (Word variations have been searched)

#10 MeSH descriptor: [Osteoarthritis, Hip]

#11 MeSH descriptor: [Osteoarthritis,Knee]

#12 MeSH descriptor: [Osteoarthritis, Spine]

#13 (“arthrosis deformans”) (Word variations have been searched)

#14 Degenerative adj2 arthritis

#15 (“degenerative joint disease”) (Word variations have been searched)

#16 (“degenerative arthritis”) (Word variations have been searched)

#17 (non-inflammatory arthritis) (Word variations have been searched)

#18 #8 or #9 or #10 or #11 or #12 or #13 or #14 or #15 or #16 or #17

#19 MeSH descriptor: [Randomized Controlled Trial] explode all trees

#20 MeSH descriptor: [Controlled Clinical Trial] explode all trees

#21 placebo*

#22 (“randomised clinical trial”) (Word variations have been searched)

#23 (trial*) (Word variations have been searched)

#24 MeSH descriptor: [Randomized Controlled Trials as Topic] explode all trees

#25 #19 or #20 or #21 or #22 or #23 or #24

#26 #7 and #18 and #25

**6. Allied and Complementary Medicine (AMED) (Ovid) <1985 to 22 September 2023>: 13 records**

**1 exp Mind body medicine/**

**2 exp Yoga/**

**3 exp Meditation/**

**4 Mind body medicine*.mp.**

**5 (Yoga* or yogi*).mp.**

**6 Meditat*.mp.**

**7 (Asana*** pranayam* or dhyan* or ashtanga or bikram or hatha or iyengar or kripalu or kundalini or vinyasa or raja or radja or bhakti or jnana or kriya* or karma or yama or niyama or pratyahara or dharana or samadhi or bandha or mudra* or chanda or sivananda).mp.

**8 1 or 2 or 3 or 4 or 5 or 6 or 7**

**9 exp Osteoarthritis/**

**10 (osteoarthr* or osteo-arthr*).mp.**

**11 Coxarthrosis.mp.**

**12 Arthrosis deformans.mp.**

**13 (Degenerative adj2 arthritis).mp.**

**14 Degenerative joint disease*.mp.**

**15 Non-inflammatory arthritis.mp.**

**16 9 or 10 or 11 or 12 or 13 or 14 or 15**

**17 (random* or factorial* or placebo* or assign* or allocat* or crossover*).tw.**

**18 (cross adj over*).tw.**

**19 (trial* and (control* or comparative)).tw.**

**20 ((blind* or mask*) and (single or double or triple or treble)).tw.**

**21 (treatment adj arm*).tw.**

**22 (control* adj group*).tw.**

**23 (phase adj (III or three)).tw.**

**24 (versus or vs).tw.**

**25 rct.tw.**

**26 RANDOM ALLOCATION/**

**27 DOUBLE BLIND METHOD/**

**28 placebos/**

**29 randomized controlled trials/**

**30 17 or 18 or 19 or 20 or 21 or 22 or 23 or 24 or 25 or 26 or 27 or 28 or 29**

**31 8 and 16 and 30**

**7. SPORTDiscus (EBSCOHost) <2004 to 22 September 2023>: 15 records**

1. SU Mind body therapy

2. SU Yoga

3. SU Meditation

4. TX Mind body therap*

5. TX Yoga* or yogi*

6. TX Meditat*

7. TX Asana* pranayam* or dhyan* or ashtanga or bikram or hatha or iyengar or kripalu or kundalini or vinyasa or raja or radja or bhakti or jnana or kriya* or karma or yama or niyama or pratyahara or dharana or samadhi or bandha or mudra* or chanda or sivananda

8. S1 or S2 or S3 or S4 or S5 or S6 or S7

9. SU Osteoarthritis

10. TX Osteoarthr* or osteo-arthr*

11. TX Coxarthrosis

12. TX Arthrosis deformans

13. TX degenerative arthritis

14. TX degenerative joint disease*

15. TX Non-inflammatory arthritis

16. S9 or S10 or S11 or S12 or S13 or S14 or S15

17. SU Randomized controlled trials

18. TX Randomized controlled trials

19. TX Double-blind studies

20. TX Single-blind studies

21. TX Random assignment

22. TX Pretest-posttest design

23. TX Cluster sample

24. TX Placebos

25. TX randomised or randomized

26. TX random*

27. TX trial*

28. S17 or S18 or S19 or S20 or S21 or S22 or S23 or S24 or S25 or S26 or S27

29. S8 and S16 and S28

**8. Web of Science <1998 to 22 September 2023>: 142 records**

**#1 ALL= (yoga* or “mind body therap*” or meditation or yogi* or asana* or pranayam* or dhyan* or meditat* or ashtanga or bikram or hatha or iyengar or kripalu or kundalini or vinyasa or raja or radja or bhakti or jnana or kriya* or karma or yama or niyama or pratyahara or dharana or samadhi or bandha or mudra* or chanda or sivananda)**

**#2 ALL= (“Osteoarthritis” or osteoarthr* or osteo-arthr* or “coxarthrosis” or degenerative arthritis or degenerative joint disease* or non-inflammatory arthritis)**

**#3 ALL= (“randomized controlled trial” or “controlled clinical trial” or “clinical trial” or “clinical trials” or placebo$ or “random allocation” or “double-blind method” or “single-blind method” or “cross-over studies”)**

**#4 ALL= (randomised or randomized or randomisation or randomisation or placebo* or (random* and (allocat* or assign*)) or (blind* and (single or double or treble or triple)))**

**#5 #3 or #4**

**#6 #1 and #2 and #5**

**9. Turning Research Into Practice (TRIP) <2014 to 22 September 2023>: 3130 records**

yoga* OR "mind body therapies" OR yogi* OR asana* OR pranayam* OR dhyan* OR meditation OR meditate OR ashtanga OR bikram OR hatha OR iyengar OR kripalu OR kundalini OR vinyasa OR raja OR radja OR bhakti OR jnana OR kriya* OR karma OR yama OR niyama OR pratyahara OR dharana OR samadhi OR bandha OR mudra* OR chanda OR sivananda AND ("osteoarthritis" OR osteoarthr* OR osteo-arthr* OR coxarthrosis OR degenerative arthritis OR degenerative joint disease* OR non-inflammatory arthritis) AND (randomised controlled trial OR randomized controlled trial OR controlled clinical trial* OR clinical trial* OR placebo* OR random*)

**10. AYUSH Research Portal (Ministry of AYUSH, Government of India): 33 records**

Medical system: yoga and naturopathy

Category: preclinical research, clinical research (Evidence grade - A, B, C) and fundamental research

Body system: musculoskeletal

Disease (English): Osteoarthrosis of hip (ICPC-L89) (ICD-M16)

Evidence grade- A:1 Evidence grade-B:0 Evidence grade-C:0

Disease (English): osteoarthrosis of knee (ICPC-L90) (ICD-M17)

Evidence grade-A:6 Evidence grade-B:23 Evidence grade-C:0

Disease (English): osteoarthrosis other (ICPC-L91) (ICD-M13, M15, M18, M19)

Evidence grade-A:1 Evidence grade-B:2 Evidence grade-C:0

**11. A Bibliography of Indian Medicine (ABIM): 1 record**

Search terms used: yoga and osteoarthritis

**12. Complementary and alternative medicine (CAM-QUEST): 61 records**

Searched for the following:

Disease pattern: pain (1155 results) to disease: chronic pain (209 results) to therapy: mind body medicine (75 results) to study design: randomized trial (33 results)

Searched for the following:

Therapy: Mind-body medicine (3708 results) to disease pattern: Musculoskeletal-/Connective tissue system (388 results) to disease: Arthritis (24 results) to study design: Randomized trial (13 results)

Therapy: Mind-body medicine (3708 results) to disease pattern: Musculoskeletal-/Connective tissue system (388 results) to disease: Arthrosis (37 results) to study design: Randomized trial (15 results)

**13. Physiotherapy Evidence Database (PeDro) <1999 to 22 September 2023>: 39 records**

Searched terms used: yoga and osteoarthritis

**14. OpenGrey (Data Archiving and Network Services) <1997 to 22 September 2023>: 0 records**

Search terms used: yoga and osteoarthritis

**15. EthOS <2009 to 22 September 2023>: 623 records**

Mind-Body Therapies or mind body therap*.mp. or meditat* or yoga* or yogi* or asana* or pranayam* or dhyan* or ashtanga or bikram or hatha or iyengar or kripalu or kundalini or vinyasa or raja or radja or bhakti or jnana or kriya* or karma or yama or niyama or pratyahara or dharana or samadhi or bandha or mudra* or chanda or sivananda or osteoarthritis or degenerative joint disease or randomized controlled trial

**16. ProQuest Dissertations and Theses <1902 to 22 September 2023>: 1051 records**

(yoga* or mind body therap* or yogi* or asana* or pranayam* or dhyan* or meditat* or ashtanga or bikram or hatha or iyengar or kripalu or kundalini or vinyasa or raja or radja or bhakti or jnana or kriya* or karma or yama or niyama or pratyahara or dharana or samadhi or bandha or mudra* or chanda or sivananda) and (osteoarthritis or osteoarthr* or osteo-arthr* or coxarthrosis or degenerative adj2 arthritis or degenerative joint disease* or non-inflammatory arthritis) and (randomised controlled trial or randomized controlled trial or controlled clinical trial* or clinical trial* or placebo* or random* or trial*)

**17. DART-Europe-e-theses portal <2005 to 22 September 2023>: 6 records**

Yoga or asana* or pranayam* or dhyan* or osteoarthritis or degenerative joint disease or non-inflammatory arthritis or RCT or randomized controlled trial

**Appendix 2: Excluded studies with reasons for exclusion**

Full-text studies excluded (n=25)

**Related to comparator (n=1)**

Buchanan DT, Vitiello MV, Bennett K. Feasibility and efficacy of a shared yoga intervention for sleep disturbance in older adults with osteoarthritis. Journal of Gerontological Nursing. 2017:10.3928/00989134-20170405-01.

**Related to outcome (n=7)**

McCaffrey R, Park K, Newman D. Chair yoga: feasibility and sustainability study with older community-dwelling adults with osteoarthritis. Holistic Nursing Practice. 2017;31(3):148-57.

Moonaz SH, Bingham CO, Wissow L, Bartlett SJ. Yoga in sedentary adults with arthritis: effects of a randomized controlled pragmatic trial. Journal of Rheumatology. 2015;42(7):1194-1202.

Middleton KR, Ward MM, Moonaz SH, Lopez MM, Tataw-Ayuketah G, Yang L, et al. Feasibility and assessment of outcome measures for yoga as self-care for minorities with arthritis: a pilot study. Pilot and Feasibility Studies. 2018;4:10.1186/s40814-018-0248-x.

Middleton KR, Ward MM, Haaz S, et al. A pilot study of yoga as self-care for arthritis in minority communities. Health and Quality of Life Outcomes. 2013;11:10.1186/1477-7525-11-55.

Park J, McCaffrey R. Strategies of recruitment and retention of older adults with osteoarthritis for a yoga intervention: clinical trial. Journal of the American Geriatrics Society. 2015; 63:32‐33.

Cheung C, Wyman JF, Savik K. Adherence to a yoga program in older women with knee osteoarthritis. Journal of Aging and Physical Activity. 2016;24(2):181-8.

Haaz S, Bingham CO, Bartlett SJ. Adherence to an 8-week yoga program for RA and OA: who drops out and why? Arthritis and Rheumatism. 2009;60:1002/art.27051

**Related to study design, e.g., not an/unclear RCT, quasi-RCT, secondary analysis (n=7)**

Park J, McCaffrey R. Chair yoga: benefits for community-dwelling older adults with osteoarthritis. Journal of Gerontological Nursing. 2012;38(5):12-25.

Park J, McCaffrey R, Newman D, Cheung C, Hagen D. The effect of Sit 'n' Fit chair yoga among community-dwelling older adults with osteoarthritis. Holistic Nursing Practice. 2014;28(4):247-57.

Park J, McCaffrey R, Dunn D, Goodman R. Managing osteoarthritis: comparisons of chair yoga, Reiki, and education (pilot study). Holistic Nursing Practice. 2011;25(6):316-26.

Kolasinski SL, Garfinkel M, Tsai AG, Matz W, Van Dyke A, Schumacher HR. Iyengar yoga for treating symptoms of osteoarthritis of the knees: a pilot study. Journal of Alternative and Complementary Medicine. 2005;11(4):689-93.

Ghasemi G, Golkar A, Marandi SM. Effects of Hata yoga on knee osteoarthritis. International Journal of Preventive Medicine. 2013;4:133-8.

Nambi GS, Shah AA. Additional effect of Iyengar yoga and EMG biofeedback on pain and functional disability in chronic unilateral knee osteoarthritis. International Journal of Yoga. 2013;6(2):123-7.

Witt CM, Michalsen A, Roll S, Morandi A, Gupta S, Rosenberg M, et al. Comparative effectiveness of a complex Ayurvedic treatment and conventional standard care in osteoarthritis of the knee - study protocol for a randomized controlled trial. Trials. 2013;14:10.1186/1745-6215-14-149.

**Ongoing RCTs (n=9)**

Study to determine effect of naturopathy and yoga treatment in patients of osteoarthritis of knee. CTRI/2010/091/001168

Dietary supplement and yogic practices for joints pain. CTRI/2020/06/025867

Effect of Ayurvedic preparation Peedanil gold with comparison to yoga module and conventional care in knee osteoarthritis patients. CTRI/2022/01/039150

Comparing effects of an online exercise plus education program to online education only in people with knee osteoarthritis: randomised controlled trial: The COPE Trial. ACTRN12620000012976

A randomised comparative effectiveness trial of YOGa and strengthening exercise for knee osteoArthritis (YOGA Trial). ACTRN12621000066886

Application of selected yoga asana on treatment of Osteoarthritis in knee. CTRI/2020/06/025912

Knee School for patients suffering from knee osteoarthritis. CTRI/2020/03/024311

Trial of 2 conservative therapies for knee arthritis patients. CTRI/2014/01/004270

Yoga for Osteoarthritis of Knee. CTRI/2022/03/040807

**Full text not available (n=1)**

Srivastava RN, Avasthi V, Srivastava SR, Raj S. Does yoga improve pain, stiffness and physical disability in knee osteoarthritis? – a randomized controlled clinical trial. Osteoarthr and Cartilage. 2015;23(2):167.
